# Light-Responsive Solid–Solid Phase Change Materials for Photon and Thermal Energy Storage

**DOI:** 10.1021/acsmaterialsau.2c00055

**Published:** 2022-09-30

**Authors:** Xiang Li, Sungwon Cho, Grace G. D. Han

**Affiliations:** Department of Chemistry, Brandeis University, 415 South Street, Waltham, Massachusetts 02453, United States

**Keywords:** phase transition, photoswitch, adamantane, solid state, energy storage, latent heat

## Abstract

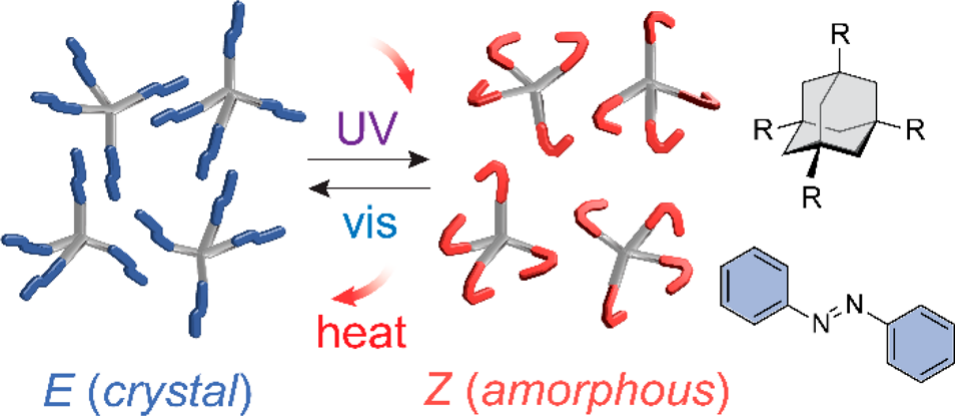

We report a series of adamantane-functionalized azobenzenes that store photon and thermal energy via reversible photoisomerization in the solid state for molecular solar thermal (MOST) energy storage. The adamantane unit serves as a 3D molecular separator that enables the spatial separation of azobenzene groups and results in their facile switching even in the crystalline phase. Upon isomerization, the phase transition from crystalline to amorphous solid occurs and contributes to additional energy storage. The exclusively solid-state MOST compounds with solid–solid phase transition overcome a major challenge of solid–liquid phase transition materials that require encapsulation for practical applications.

Molecular solar thermal (MOST) energy storage compounds that store photon energy in strained chemical bonds upon photoisomerization have emerged as a novel material that harnesses solar energy and releases the stored energy as heat on demand.^1−3^ MOST compounds are generally photoswitches that undergo structural isomerization between the ground-state and metastable-state isomeric forms, and the energy difference between the two isomers, i.e., isomerization energy (Δ*H*_iso_), is stored in the system. Among various photoswitch designs known to date, including norbornadienes,^4−6^ dihydroazulenes,^7−9^ hydrazones,^10−13^ spiropyrans,^14,15^ donor–acceptor Stenhouse adducts,^16,17^ and fulvalene diruthenium complexes,^18,19^ azobenzene and its derivatives have been extensively explored for MOST energy storage, due to the ease of synthesis and derivatization,^20,21^ tunable optical properties and thermal half-lives (*t*_1/2_),^22−26^ and reversible isomerization over many cycles with little degradation.^27^ Azobenzene derivatives also undergo large structural and polarity changes upon *E–Z* isomerization, which often results in the phase transition between the crystalline *E* and liquid *Z* isomers. The photoinduced phase transition favorably increases the total energy storage density; in addition to Δ*H*_iso_, phase transition energy is stored during the *E* → *Z* isomerization.^23,28,29^

This strategy of incorporating solid–liquid phase transition to isomerization has been widely investigated for azobenzenes and azoheteroarenes,^23,29,30^ typically functionalized with alkyl chains (Figure 1a). These phase-transition MOST compounds exhibit large energy densities over 300 J/g, which opens up opportunities to utilize them for practical energy applications. However, such solid–liquid phase transition has a couple of critical challenges that need to be addressed for successful applications. First, the potential leakage of organic liquid increases the risk of combustion, which is also considered as a major limitation for common organic phase change materials such as paraffins^31^ and fatty acids^32^ used for thermal energy storage. Strategies including the encapsulation of liquid phase have been developed, but the use of encapsulating polymers and carbonaceous materials^33−36^ that do not contribute to energy storage lowers the overall gravimetric energy density of the system. Second, the UV-induced liquefaction (Figure 1a) is often unsuccessful at room temperature.^37,38^ Due to the strong π–π interactions between the azobenzenes and the van der Waals interactions among the alkyl chains in the crystalline phase, the conformational freedom of azobenzene is limited, and the large geometrical change of azobenzene is not accommodated in the crystals. Therefore, the initial heating and melting of the *E* crystals are necessary to facilitate the *E* → *Z* isomerization and the formation of the *Z* liquid, which limits the temperature range within which photon energy can be stored in such compounds.

**Figure 1:**
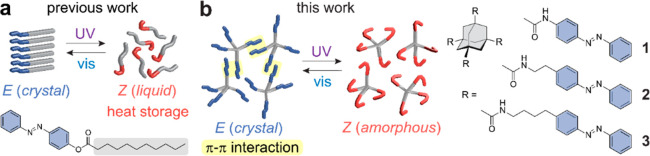
(a) MOST energy storage through photoinduced isomerization and solid–liquid phase transition of alky-functionalized azobenzene. (b) Reversible solid–solid phase transition of adamantane-functionalized azobenzene derivatives and their chemical structures.

In order to address these prominent challenges of the solid–liquid phase transition photoswitches, we investigate a new design of MOST materials that undergo solid–solid phase transition at room temperature (Figure 1b). The strategy involves the placement of 3D molecular separators in the solid matrix of azobenzene switches, which reduces the packing density of photochromes and allows the isomerization to occur at room temperature in the crystalline phase. The choice of adamantane as a 3D separator enables the tetrahedral placement of azobenzenes and lowers the degree of intermolecular π–π interactions. The covalent linkage between the adamantane core and azobenzene groups is essential; the noncovalent mixing between the azobenzene and adamantane units would result in phase separation and create close-packed azobenzene crystals that cannot switch at room temperature. The distance between azobenzene and adamantane can be modulated by adding alkyl linkers, which tunes the degree of intermolecular interactions and leads to the formation of different crystalline phases.

Three-dimensional (3D) star-shaped azobenzene tetramers have been reported by Baroncini, Credi, and co-workers,^39,40^ based on a rigid tetra(azobenzene)methane scaffold. The shape-persistent tetramers were designed to generate microporosity, and the optically controlled caption of CO_2_ within the porous materials was evaluated. On the other hand, their ability to store photon energy was not probed. A triptycene scaffold was also used to arrange azobenzenes in a 3D space, reported by Wegner and co-workers,^41^ to study excitonic coupling between the photochromes, while their potential for energy storage has not been explored. In general, porous materials with a large pore volume are not desired for MOST energy storage due to their low volumetric energy densities. Therefore, we aim to create nonporous materials composed of azobenzenes and 3D molecular separators that are linked through flexible bonds, as opposed to the reported porous materials consisting of rigid tetramers. To the best of our knowledge, harnessing a 3D spatial arrangement of azobenzenes for solid-state MOST energy storage has remained unexplored. Herein, we report a unique class of MOST compounds that undergo solid–solid phase transition upon isomerization to store both isomerization energy and phase transition energy.

The optical properties of compounds **1**–**3** were examined both in solution and in the solid state, as shown in Figure 2a and b for compound **2**. Other compounds exhibit similar absorption features (Figure S1). The thermal half-lives of compounds **1**–**3** in solution state were measured in DMSO and vary from 1 to 6 days (Figure S2, Table S1). The absorption spectra of the compounds show π–π* transition centered around 340 nm and n−π* around 430 nm, retaining the characteristics of unsubstituted azobenzene. At the photostationary state (PSS) under irradiation at 340 nm, ∼95% *Z* is obtained in solution, whereas slightly less than 50% *Z* is acquired in thin films for all compounds (Figures S3–S5). An average thickness of ∼1 μm of the films was measured by profilometry (Figures S6–S8). The suboptimal PSS ratio for solid-state photoswitches is attributed to the significant overlap between the optical absorption of *E* and *Z* isomers, which leads to a small penetration depth of incident light through the condensed phase materials. However, compared to the films of unsubstituted azobenzene at a comparable thickness, which do not allow any solid-state switching (Figure S9), ∼50% conversion obtained for compounds **1**–**3** is substantial. This verifies the role of the 3D molecular separator, the adamantane unit, in increasing the conformational freedom of photoswitches in the crystalline solid. The *Z* → *E* reversion promoted by the irradiation at 430 nm is more complete in thin films (100%) than in solution (83%), which is attributed to the different polarity of the switching media.^42,43^ In thin films, the alternating irradiation at 340 and 430 nm successfully switches the azobenzene over 10 cycles without any noticeable degradation, confirming its excellent photostability as well as morphological stability that ensures the reversible switching of azobenzene in the confined space within the solid state (Figure 2c). Both compounds **1** and **3** showed similar cyclability as **2** (Figure S10).

**Figure 2:**
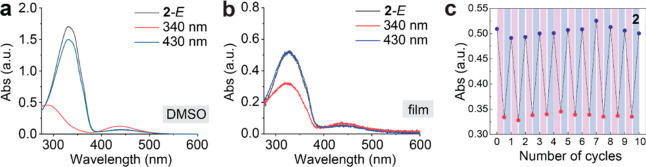
(a) UV–vis absorption spectra of compound **2** as prepared (black), after irradiation at 340 nm (red), and 430 nm (blue) measured in DMSO solutions (0.02 mM). (b) UV–vis absorption spectra of compound **2** measured in thin films. (c) Absorption changes of compound **2** at 330 nm upon the repeated irradiation at 340 nm (purple filled area) and 430 nm (blue filled area) in thin films.

Compounds **1**–**3** appear as an orange powder in *E* isomeric forms and become a darker orange-red powder when switched to *Z* isomers (Figure 3 insets). These condensed phase materials were analyzed by differential scanning calorimetry (DSC) to understand their phase transitions and energy storage capacities (Table 1). Compound **1-***E* with the shortest amide linkage between the adamantane separator and azobenzene groups displays no melting or crystallization features during the heating and cooling cycles of DSC (Figure 3a). Heating the compound above 260 °C results in thermal decomposition, which infers that compound **1-***E* has a very high melting point that is difficult to measure under atmospheric pressure. **1-***Z* shows an exotherm indicating the *Z → E* thermal reversion, followed by a smaller exotherm above 200 °C. The first exothermic peak displays a shoulder above 100 °C, which indicates a partial crystallization of *E* isomer that is generated upon the *Z* → *E* isomerization. The second exotherm above 200 °C shows further crystallization of the *E* isomer from a partially amorphous state, which was not completed at the lower temperature during the reversion, in the mixture of *Z* and *E* isomers. Due to the high melting point of **1**-*E*, the subsequent melting after crystallization could not be recorded. Instead, we confirmed the *Z* → *E* thermal reversion after DSC by ^1^H NMR (Figure S11). Both the isomerization energy (Δ*H*_iso_) and phase transition energy (Δ*H*_c_) are stored in the amorphous *Z* isomer and released upon the triggering of *Z → E* reversion and simultaneous crystallization.

**Table 1:** Thermal Parameters of Compounds **1**–**3** Measured by DSC^a^

	***E***			*Z* → *E*				
	*T*_m_ (°C)	*T*_c_ (°C)	Δ*H*_c_ (J/g)	T_iso_ (°C)	Δ*H*_iso_ (J/g)	Δ*H*_iso_ (kJ/mol)	Δ*H*_total_ (J/g)	Δ*H*_total_ (kJ/mol)
**1**	–	–	–	101	118	121	139	143
**2**	239	195	34	99	107	122	141	161
**3**	202	122	31	95	87	109	118	149

a *T*_m_: melting temperature, *T*_c_: crystallization temperature, Δ*H*_c_: crystallization energy, T_iso_: isomerization peak temperature, Δ*H*_iso_: *E–Z* isomerization energy, Δ*H*_total_: total energy storage, −: unable to measure due to thermal decomposition.

**Figure 3:**
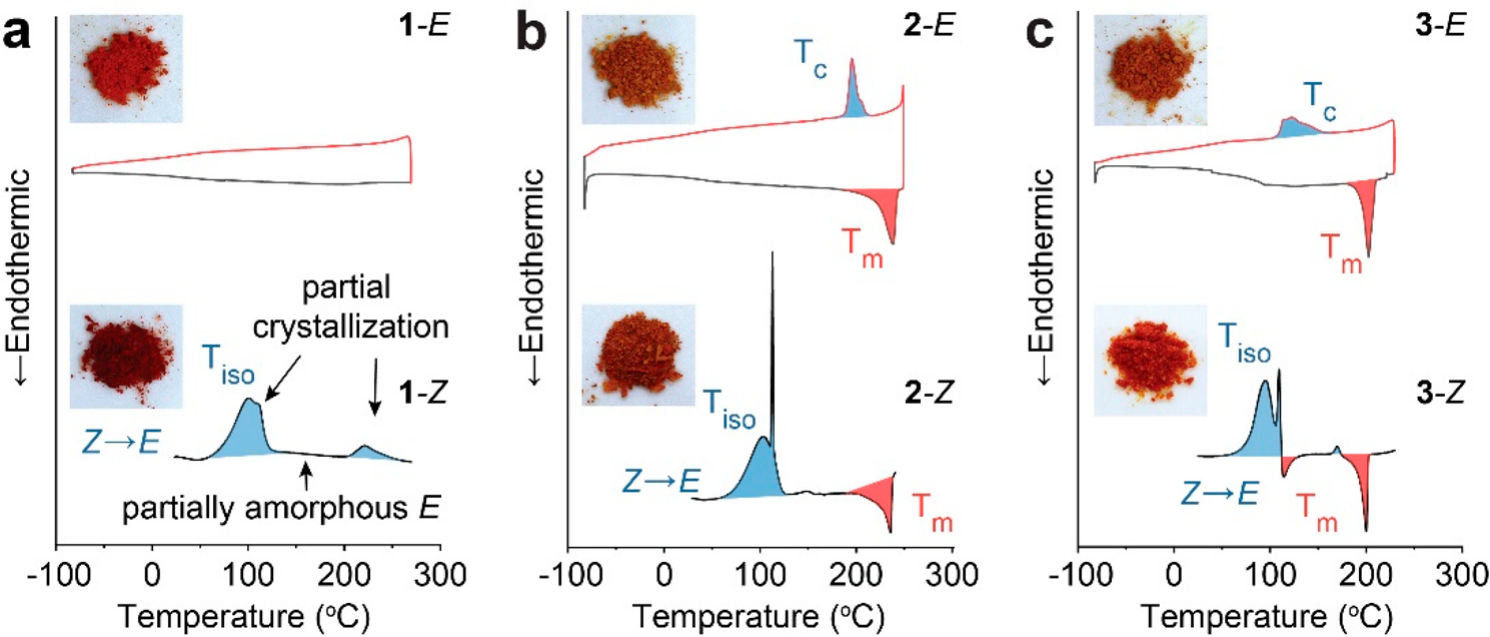
DSC plots of compounds (a) **1**, (b) **2**, and (c) **3** in the *E* and *Z* isomeric forms. *E* isomers were heated (black curve) then cooled (red curve) to between −90 and >250 °C, and *Z* isomers were heated from room temperature to monitor *Z* → *E* thermal reversion. *T*_iso_: isomerization peak temperature, *T*_c_: crystallization temperature, *T*_m_: melting temperature.

In Figure 3b, compound **2** shows clear melting and crystallization points as an *E* isomer, indicating the crystalline nature of *E*. On the other hand, the heated *Z* isomer shows a convoluted exotherm representing *Z* → *E* isomerization (broad peak) and subsequent crystallization (sharp peak). The continued heating leads to a melting peak that is identical to that of **2**-*E*, verifying the generation and crystallization of the *E* isomer upon heating of **2**-*Z*. Compound **2** has an ethyl linker between the adamantane and azobenzene groups. We hypothesize that the flexibility of alkyl linkers contributes to reducing the intermolecular π–π interactions among azobenzene units and decreases the melting and crystallization points of *E* isomers. Compound **3** bearing the longest butyl linker shows the lowest melting and crystallization points among the *E* isomers of compounds **1**–**3**, corroborating the hypothesis (Table 1). Interestingly, compound **3**-*Z* displays an exotherm of *Z* → *E* isomerization and an immediate crystallization of *E*, followed by 1) a minor melting peak, 2) a minor crystallization peak, and 3) a large endotherm representing the melting of **3**-*E* crystal (Figure 3c). Due to the larger disorder rendered by the long alkyl linkers, the *E* isomer has polymorphs including a minor crystalline phase with a lower melting point and a major phase with a higher melting point. The polymorphism of compound **3**-*E* is also manifested in its second and third DSC cycles (Figure S12c, f), showing two sets of melting and crystallization points. The second and third cycles of DSC for compounds **1**–**3** are illustrated in Figure S12.

Based on the integrated exotherms associated with *Z → E* isomerization and the crystallization of *E*, we can determine the energy storage capacity of compounds (Δ*H*_total_). For compound **1**, the isomerization energy (Δ*H*_iso_) was estimated by the integration of the exotherm at 101 °C, which partially overlaps with a minor crystallization peak and is well separated from the subsequent crystallization peak. For compounds **2** and **3**, due to the more significant convolution of the isomerization peak and *E* crystallization peak, their Δ*H*_iso_ values were estimated from the difference between the Δ*H*_total_ and the crystallization energy of *E* isomers (Δ*H*_c_), obtained separately from the cooling of molten *E* isomers. Compounds **1** and **2** exhibit similar gravimetric Δ*H*_total_ of 139 and 141 J/g, while that of compound **3** is lower (118 J/g), which is attributed to the large molecular weight of compound **3**. Δ*H*_total_ per molecule is similar between compounds **1** and **3** (143 and 149 kJ/mol), and compound **2** is the largest (161 kJ/mol). It highlights the significance of solid–solid phase transition in increasing the overall energy storage of the MOST compounds. Upon *Z → E* reversion, compound **2** immediately crystallizes, releasing the crystallization energy, in addition to Δ*H*_iso_ of the molecule. The crystallization process of compound **1** is not as facile, and such process of compound **3** overlaps with the subsequent melting of a minor crystalline phase of *E* isomer. It concludes that the mechanism of energy storage through *E*–*Z* isomerization and the associated phase transition is identical across three compounds, and compound **2** with the most distinct phase transition as shown in the DSC plot (Figure 3b) is the most desirable solid-state MOST candidate for energy storage applications.

The X-ray diffraction patterns of *E* and *Z* isomers of compound **3** (Figure 4a) corroborate the phase transition observed from the DSC. At room temperature, the *E* isomer shows multiple peaks of diffraction, indicative of a crystalline phase. The *Z* isomer, on the other hand, exhibits broad features resulting from an amorphous phase. Other compounds also show a similar crystalline (*E*) and amorphous (*Z*) nature by diffraction (Figure S13). The gas adsorption measurement of all three compounds revealed small BET surface areas ranging from 5 to 57 m^2^/g, verifying their nonporous structures (Figure S14, Table S2). Figure 4b and c summarizes the key findings of this work; the crystalline *E* isomers undergo the UV-induced isomerization and phase transition to an amorphous *Z* phase, storing photon and thermal energy. The amorphous *Z* isomers are triggered by visible light irradiation to revert to the crystalline *E*, releasing the stored energy as heat. The intermediate, amorphous *E* state is difficult to isolate in experiments, due to the immediate crystallization of *E* when generated from the isomerization of *Z*, as shown in Figure 3. The overall energy storage in amorphous *Z* is the combination of Δ*H*_iso_, measured in the amorphous phase, and Δ*H*_c_ of the *E* isomer, both of which were assessed by DSC. Notably, the contribution of phase transition to the total energy storage is considerably small compared to that of the isomerization energy. This is in contrast to solid–liquid phase transition photoswitches (Figure 1a) for which the relative magnitude of isomerization energy and phase transition energy is similar due to the long alkyl chains with substantial van der Waals interactions increasing the phase change energy.

**Figure 4:**
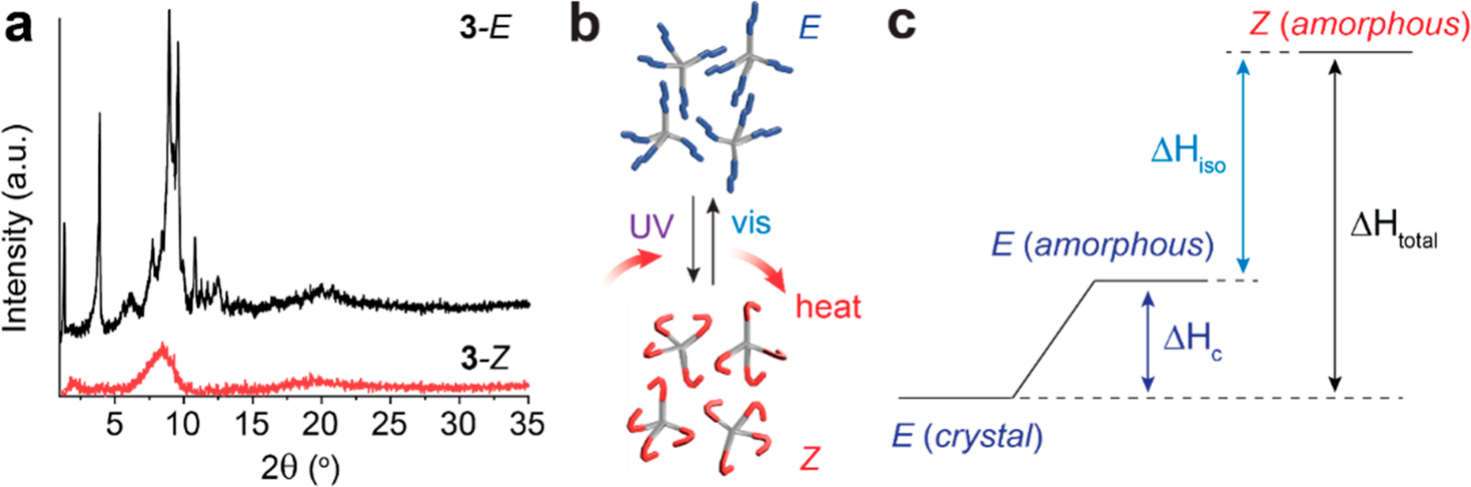
(a) XRD patterns of compound **3** as *E* and *Z* isomers. (b) Schematic illustration of the heat storage and release based on the UV and visible light-induced isomerization and solid–solid phase transition. (c) Energy diagram showing the relative energy levels of *E* in the crystalline and amorphous phase and the amorphous *Z* isomer. Δ*H*_c_: crystallization energy, Δ*H*_iso_: *E–Z* isomerization energy, Δ*H*_total_: total energy storage in the *Z* isomer.

For the new compounds, the presence of the adamantane separator reduces the intermolecular interactions among the azobenzene moieties, which allows them to undergo a large geometrical change within the crystalline phase without the need for melting the solid before irradiation. This series of MOST compounds that undergo solid–solid phase transition upon switching is unique; previously reported solid-state MOST materials, such as azobenzene-functionalized polymers^37,44−46^ and nanocarbons,^27,47,48^ as well as bulky-group-substituted azobenzenes,^49^ exhibit no phase transition upon photoirradiation, preserving the amorphous nature of the materials. Metal–organic frameworks incorporating photoswitches, on the other hand, typically maintain their crystallinity, despite pore structure changes.^50−53^

In summary, we have developed a series of azobenzene derivatives that are covalently linked to an adamantane unit that serves as a 3D molecular separator to reduce the close packing of azobenzenes in a crystalline phase. The design of nonporous solid materials enabled the facile *E*–*Z* switching of azobenzene in the confined space, due to the spatial arrangement of photochromes, which resulted in the crystalline-to-amorphous phase transition upon photoswitching. MOST energy storage materials that harness both the isomerization energy of photoswitches as well as their phase transition energy, while maintaining a solid state, would successfully circumvent the need for liquid encapsulation and have important implications in practical solar energy storage devices.

## Supporting Information Available

Experimental details, synthetic procedures, NMR spectra of *E* and *Z* isomers of compounds **1**–**3**, UV–vis spectra of compounds **1** and **3** in solution and thin films, thermal half-life measurements, thickness measurements of thin films, DSC plots, PXRD data, and gas adsorption properties (PDF)
