# Supplementary material for: Light-Responsive Solid–Solid Phase Change Materials for Photon and Thermal Energy Storage
Source: ACS Mater Au. 2022 Sep 30;3(1):37–42. doi: 10.1021/acsmaterialsau.2c00055 (PMC9838185; doi:10.1021/acsmaterialsau.2c00055)
Supplement: Supplementary file 1 — mg2c00055_si_001.pdf [file mg2c00055_si_001.pdf]

Light-Responsive Solid-Solid Phase Change Materials for Photon and Thermal Energy Storage

Xiang Li, Sungwon Cho, and Grace G. D. Han*

Department of Chemistry, Brandeis University, 415 South Street, Waltham, MA, 02453, USA

Email: gracehan@brandeis.edu

Table of Contents

1. General methods 3

UV-Vis absorbance spectroscopy... 3

Differential scanning calorimetry (DSC)... 3

Preparation of *Z*-isomer samples for DSC measurement 4

Thin film experiments.. 4

Powder X-ray diffraction (PXRD) measurements 4

Gas adsorption measurements..4

2. Synthesis procedures.. 5

3. Photoswitching properties in solution and solid state 16

4. Thermal half-life measurements in DMSO solution 17

5. Percentage of isomers at photostationary state 18

6. Thickness measurements of thin films... 21

7. Photoswitching in solid state (continued) …... 24

8. ^{1}H NMR of **1**-*Z* after DSC measurements 25

9. DSC plots.. 26

10. PXRD data...27

11. Gas adsorption properties.. 27

12. References... 28

1. General Methods

All reagents and starting materials were purchased from commercial vendors and used as supplied unless otherwise indicated. All dry solvents were obtained from the solvent system and dry triethylamine was distilled from CaH_2 and freshly used. All reactions were monitored by thin-layer chromatography (TLC) using Merck silica gel 60 F254 plates (0.25 mm). TLC plates were visualized using UV light (254 nm). Silica column chromatography was performed using Merck Silica Gel 60 (230–400 mesh). Deuterated solvents were purchased from Cambridge Isotope Laboratories, Inc. and used as received. ^{1}H NMR and ^{13}C NMR were recorded on a Varian INOVA 400 spectrometer at 400 MHz. Chemical shifts are quoted in ppm relative to tetramethylsilane (TMS) using the residual solvent peak as the reference standard. ESI mass spectra were obtained on a Waters Quattro II ESI mass spectrometer.

UV-Vis Absorbance Spectroscopy

UV−Vis adsorption spectra of compounds 1-3 were obtained with a Cary 60 Bio UV−vis spectrophotometer in a UV Quartz cuvette with a path length of 10 mm. Compounds were dissolved in DMSO. The UV−vis absorption was first recorded in dark for 3-5 min, then samples were irradiated with a specified wavelength until no change in their absorbance was observed. Samples were irradiated with a series of Thorlabs LEDs: M340L4 (340 nm, 2.22 μW/mm^2, 60 mW), M365LP1 (365 nm, 21.0 μW/mm^2, 2000 mW), and M430L4 (430 nm, 35.3 μW/mm^2, 600 mW). The condensed state UV-vis adsorption spectra of compounds 1-3 were collected with the same spectrophotometer using ultra-thin films (0.7-1.3 μm thick).

Thermal Half-life Measurements

Solutions of compounds 1-3 in DMSO were prepared and then irradiated at 365/340 nm to obtain a *Z*-rich state. The solutions were then heated at elevated temperatures in dark. The change in the concentration of the *E* isomer as a function of time was monitored, and the half-lives were obtained based on Eyring-Polanyi plots.

Differential Scanning Calorimetry (DSC) DSC analysis was conducted on a DSC 250 (TA Instruments) with an RSC 90 cooling component. All samples were heated to ~250 °C and cooled to −90 °C before reheating. *E* isomers of the compounds were heated and cooled at a rate of 10 °C/min, while *Z* isomers were at a rate of 5 °C/min to distinguish between isomerization and

phase transition.

Preparation of *Z*-isomer Samples for DSC Measurements

Z isomers were obtained by dissolving each *E* isomer in dichloromethane and irradiating the sample with an appropriate wavelength of light until the photostationary state was reached. *Z*-rich samples were concentrated, dried under high-vacuum, and then transferred to DSC pans for analysis. ^{1}H NMR spectra were taken before the DSC measurements to determine the percentage of *Z* isomers in the samples.

Thin Film Experiments

Thin-film samples were prepared by drop-casting 100 µL of 0.01 M DMSO solution of *E* isomers on a clean glass slide (2.5 x 2.5 cm^2) and heating them on a hot plate at 100 °C until the solvent completely evaporated. Then the sample was slowly cooled to room temperature. The temperature was controlled using a VWR Advanced hot plate stirrer. The thickness of the films was then measured using Zeta-20 Optical Profilometer.

E-*Z* reversible photoisomerization of thin film samples of compounds 1-3 was achieved by the direct irradiation of 365/340 nm LEDs for 1-3 h at room temperature. *Z*-*E* photoisomerization was achieved by the irradiation with 430 nm LED for 15 min at room temperature.

The UV-Vis spectra of thin films were obtained from the center of the films, and the percentage of *Z* isomers in the film was obtained by the ^{1}H-NMR spectroscopy of the dissolved central part of films.

Powder X-ray Diffraction (PXRD) Measurements

X-ray powder diffraction measurements in the 2θ range of 0-35° (step size, 0.014°; time/step, 20 s; 0.04 rad soller; 40 mA × 60 kV) were collected on a PANalytical Empyrean diffractometer equipped with an GaliPIX3D line detector and in Bragg-Brentano geometry, using Mo-Kα radiation (λ=0.7093187 Å) without a monochromator. Around 5 mg of the samples were loaded into capillary tubes (outer diameter=0.7 mm), and the measurements were carried out on the capillary spinner.

Gas Adsorption Measurements N_2 adsorption-desorption isotherms of all samples were acquired

at 77 K using a Micrometritics analyzer 3Flex at center for nanoscale systems (CNS) at Harvard. All gas adsorption measurements were carried out by first degassing samples at 150 °C for *E* isomers or at room temperature in dark for *Z* isomers overnight to minimize thermal reversion. The Brunauer, Emmet, Teller (BET) model was used to evaluate the specific surface areas on the N_2 adsorption isotherm collected at 77 K.

2. Synthetic Procedures

The compound **S1-S4** were synthesized according to reported procedures, and their identity confirmed by comparing the obtained ^{1}H NMR spectra with the spectra published in the literature.[1–4]

Scheme S1. An overview of the synthetic pathway to obtain adamantane precursors.

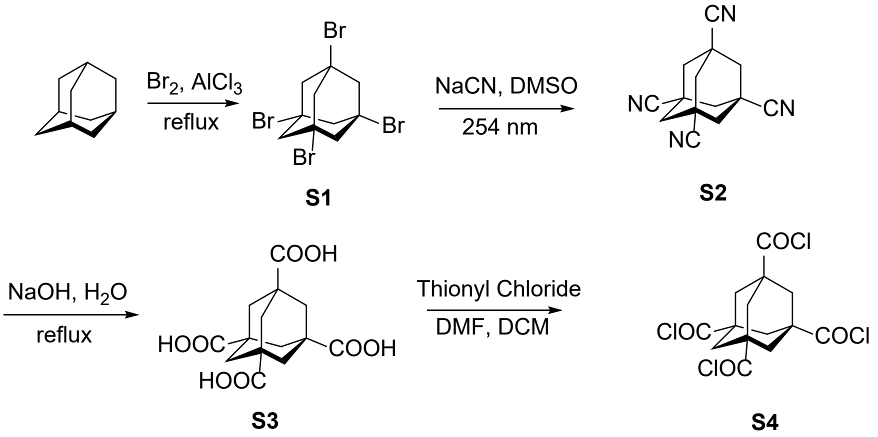

Scheme S2. Synthesis of compound **1**

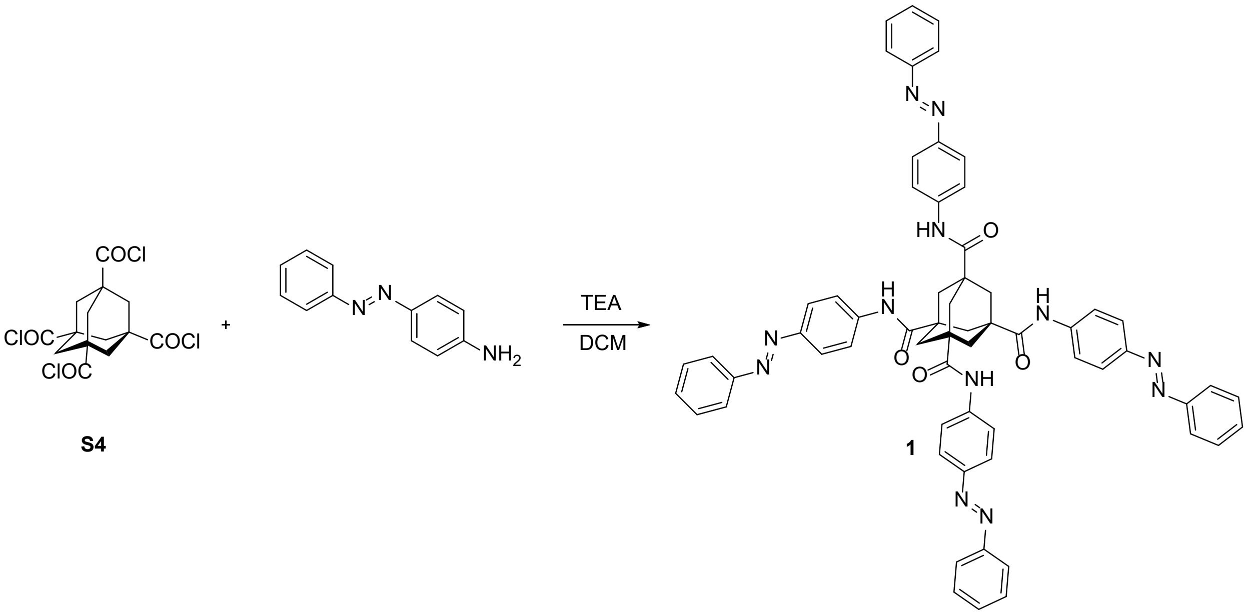

1: 4-aminoazobenzene (198.4 mg, 1.0 mmol, 5.1 eq.) was added to a 25 mL flamed-dried Ar-filled RBF with 2 mL dry DCM, and 0.15 mL dry triethylamine was added to the above solution. After stirring for 10 min, **S4** (76.4 mg, 0.2 mmol, 1 eq.) in 5 mL dry DCM was added dropwise to the reaction mixture within 15 min. Then the mixture was stirred under argon atmosphere overnight. The solution was washed with brine and extracted with DCM 3 times. The solvent was removed under vacuum and the reaction mixture was washed with chloroform to remove extra 4-aminoazobenzene. Pure product compound **1** was obtained with a yield of 83.4 mg, 40.5%. ^{1}H NMR (400 MHz, DMSO-d_6) δ 9.83 (s, 4H), 7.98 (d, J=12, 8H), 7.93 (d, J=12, 8H), 7.87 (d, J=8, 8H), 7.58 (m, 12H), 3.33 (s, H_2O), 2.30 (s, 12H) ppm. ^{13}C NMR (100 MHz, DMSO-d_6) δ 174.65, 151.99, 147.23, 130.98, 129.34, 123.38, 122.27, 120.30, 43.40, 37.69 ppm. HRMS: ESI-MS: *m/z* found [M-H+] for $C_{62}H_{52}N_{12}O_4^+$ 1029.4277 (calcd. 1029.43).

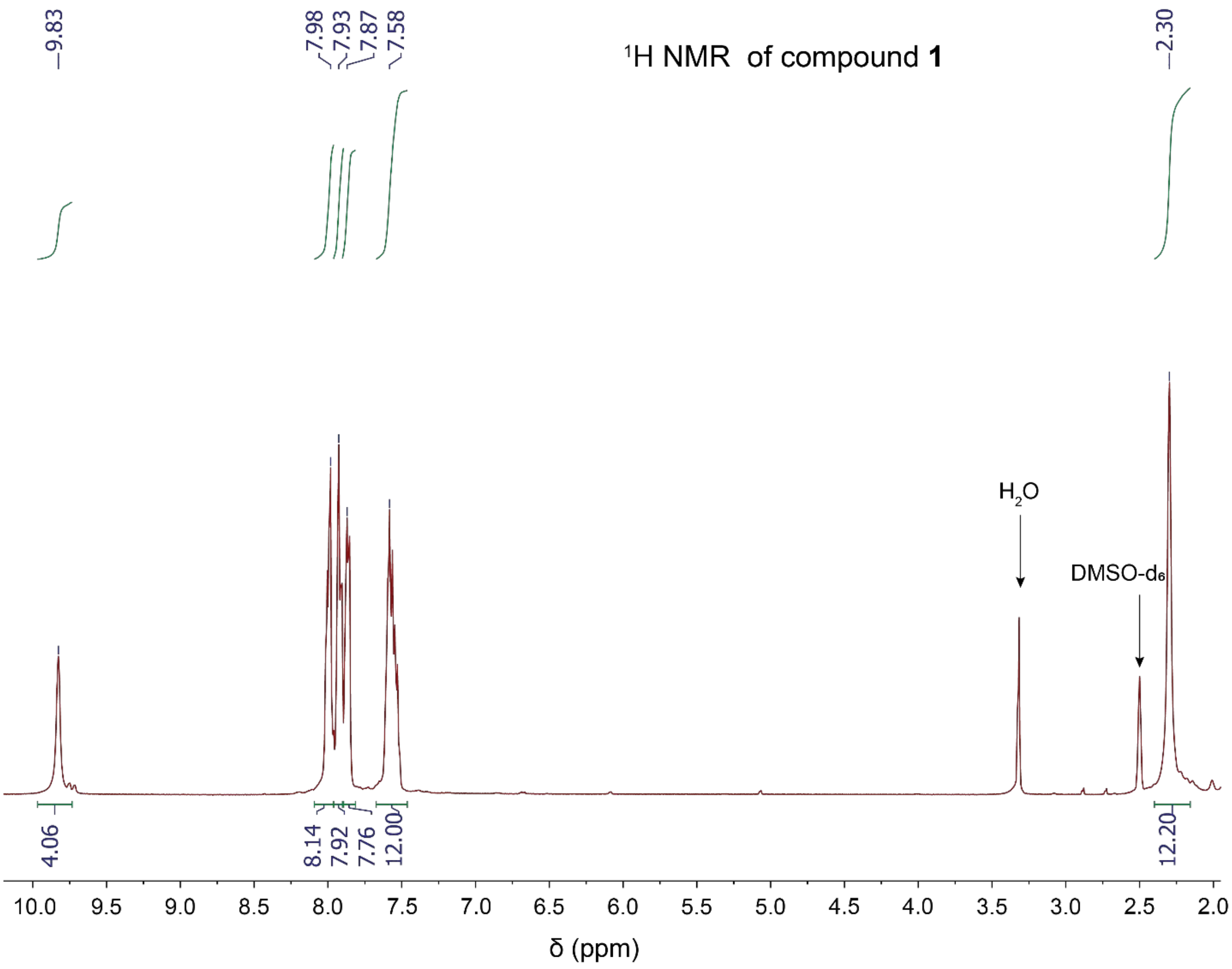

9.83
7.98
7.93
7.87
7.58
^{1}H NMR of compound **1**
2.30
H2O
DMSO-d6
4.06
8.14
7.92
7.76
12.00
12.20
10.0
9.5
9.0
8.5
8.0
7.5
7.0
6.5
6.0
5.5
5.0
4.5
4.0
3.5
3.0
2.5
2.0
δ (ppm)

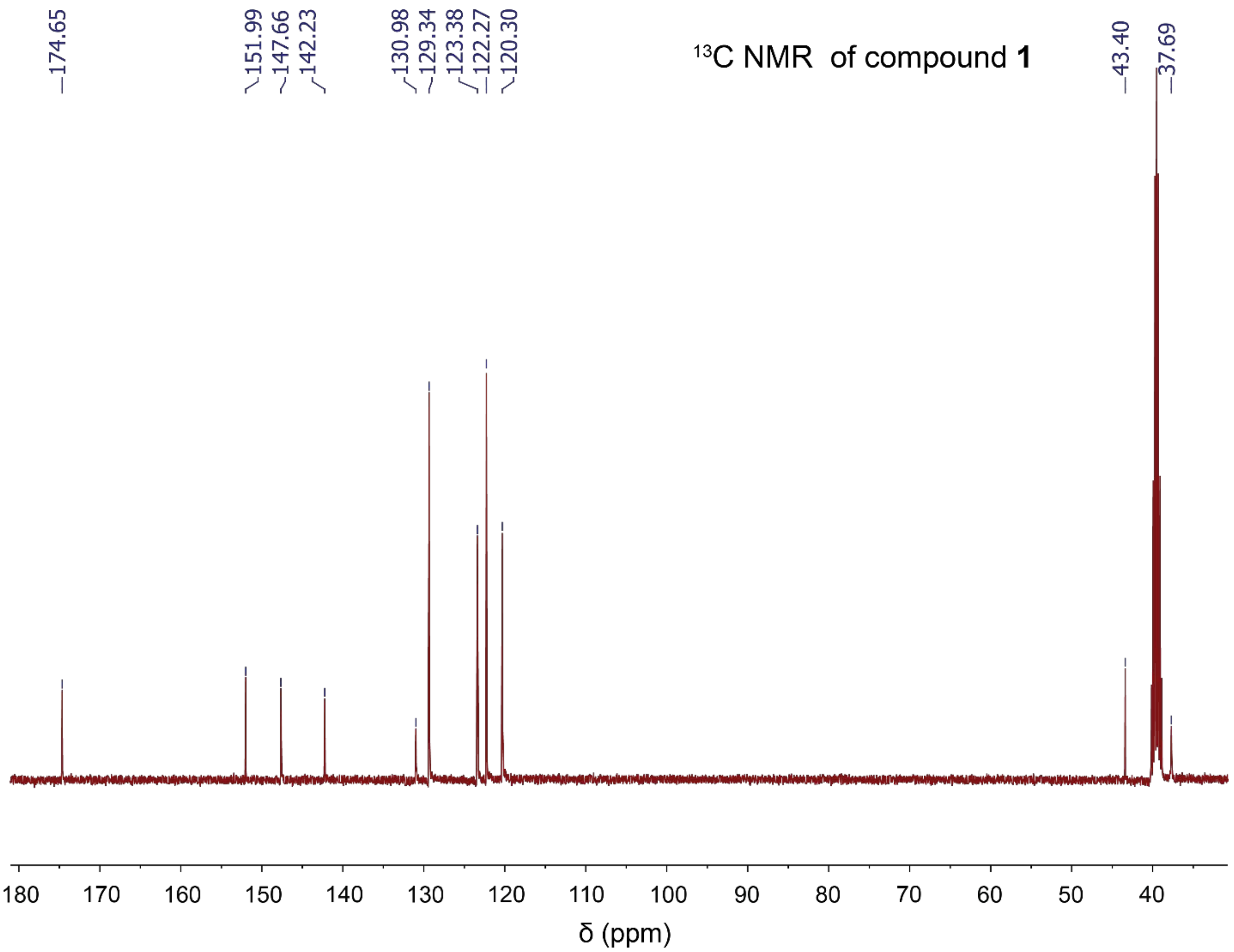

174.65
151.99
147.66
142.23
130.98
129.34
123.38
122.27
120.30
^{13}C NMR of compound **1**
43.40
37.69
180
170
160
150
140
130
120
110
100
90
80
70
60
50
40
δ (ppm)

Scheme 3. Synthesis of compound **2**

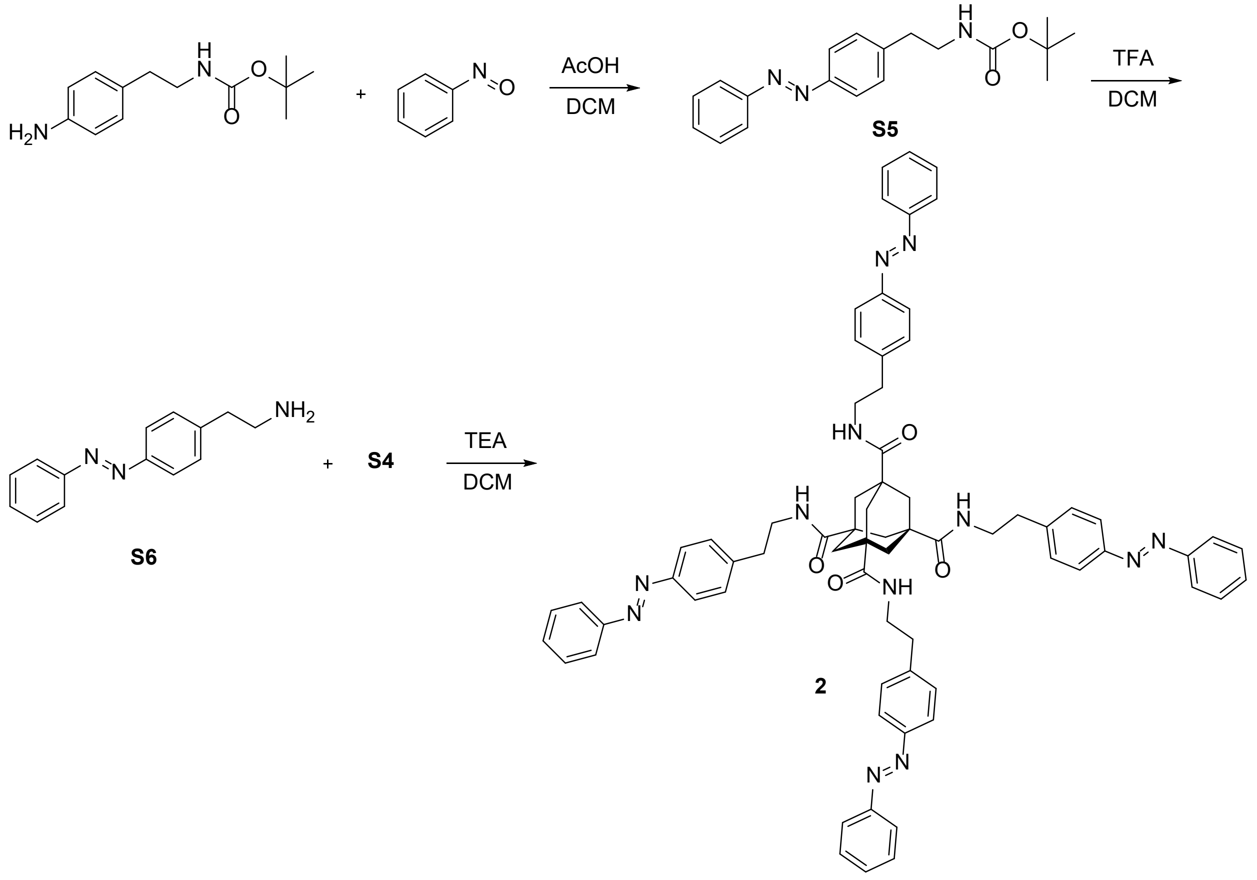

S5: 4-[2-(Boc-amino)ethyl]aniline (385.4 mg, 1.63 mmol, 1 eq.) was dissolved in 5 mL DCM in a 25 mL RBF, and nitrosobenzene (196.7mg, 1.84 mmol, 1.1 eq.) was added to the solution. Then 1 mL of concentrated acetic acid was added dropwise to the above solution. The solution was stirred at room temperature overnight. The reaction mixture pH was adjusted with sat. $NaHCO_3$ solution until a pH of ~8 was reached. Then the solution was diluted with DCM and washed with sat. $NaHCO_3$ solution and brine. The organic layer was combined and dried with anhydrous $MgSO_4$. The solvent was removed under vacuum, and the resulting crude product was purified by column chromatography (SiO_2, Hexane:EtOAc, 85:15) to yield a red solid (389.8 mg, 73.5% yield). ^1H NMR (400 MHz, $CDCl_3$) δ 7.92 (d, J=7.6 Hz, 2H), 7.88 (d, J=8 Hz, 2H), 7.52 (t, J=6.8 Hz, 2H), 7.47 (t, J=7.2 Hz, 1H), 7.35 (d, J=8.4, 2H), 4.56 (s, 1H), 3.42 (t, J=6.4, 3H), 2.89 (t, J=6.8, 3H), 1.44 (s, 9H) ppm.

S6: **S5** from the previous step was dissolved in DCM, and 1.4 mL of trifluoroacetic acid was added to the solution. The reaction mixture was stirred at room temperature for 1 h. The pH of the reaction mixture was then adjusted to a pH of ~8 by adding sat. $NaHCO_3$ solution. The solution was diluted

with DCM and washed with sat. $NaHCO_3$ solution and brine. The organic layer was combined and dried with anhydrous $MgSO_4$. The solvent was removed under vacuum and the pure product was obtained as a red solid (222.7 mg, 82.5% yield). ^1H NMR (400 MHz, CD_3OD) δ 7.88 (d, J=9.2 Hz, 2H), 7.88 (d, J=11.2 Hz, 2H), 7.54 (t, J=6.8 Hz, 2H), 7.51 (t, J=6.4 Hz, 1H), 7.43 (d, J=8.4, 2H), 2.94 (t, J=6.4, 2H), 2.86 (t, J=7.2, 2H) ppm.

2: The synthesis procedure of compound **2** was the same as compound **1**. And the resulting crude product was purified by column chromatography (SiO_2, DCM:MeOH, 98:2) to yield a yellow solid (94.3 mg, 41.3% yield). ^1H NMR (400 MHz, DMSO-d_6) δ 7.82 (t, J=8 Hz, 16H), 7.65 (t, J=5.2 Hz, 4H), 7.53 (d, J=5.2 Hz, 12H), 7.40 (d, J=7.6 Hz, 8H), 5.75 (s, DCM), 3.35 (m, 8H + H_2O), 2.83 (t, J=6.8 Hz, 8H), 2.08 (s, Acetone), 1.73 (s, 12H) ppm. ^{13}C NMR (100 MHz, DMSO-d_6) δ 175.42, 151.90, 150.40, 143.64, 131.18, 129.71, 122.49, 122.34, 41.76, 34.94, 30.62 ppm. HRMS: ESI-MS: *m/z* found [M-H+] for $C_{70}H_{68}N_{12}O_4^+$ 1141.5546 (calcd. 1141.55).

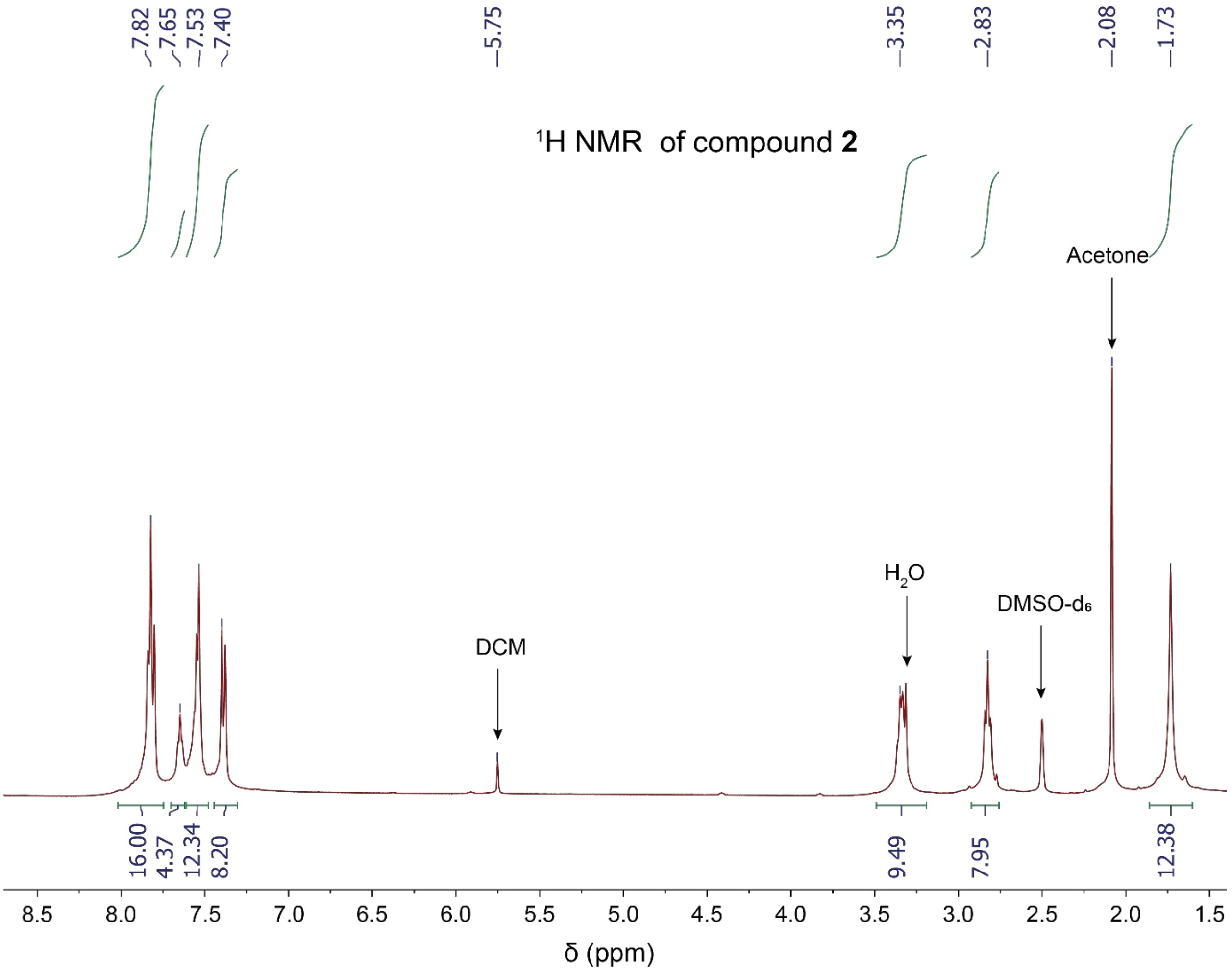

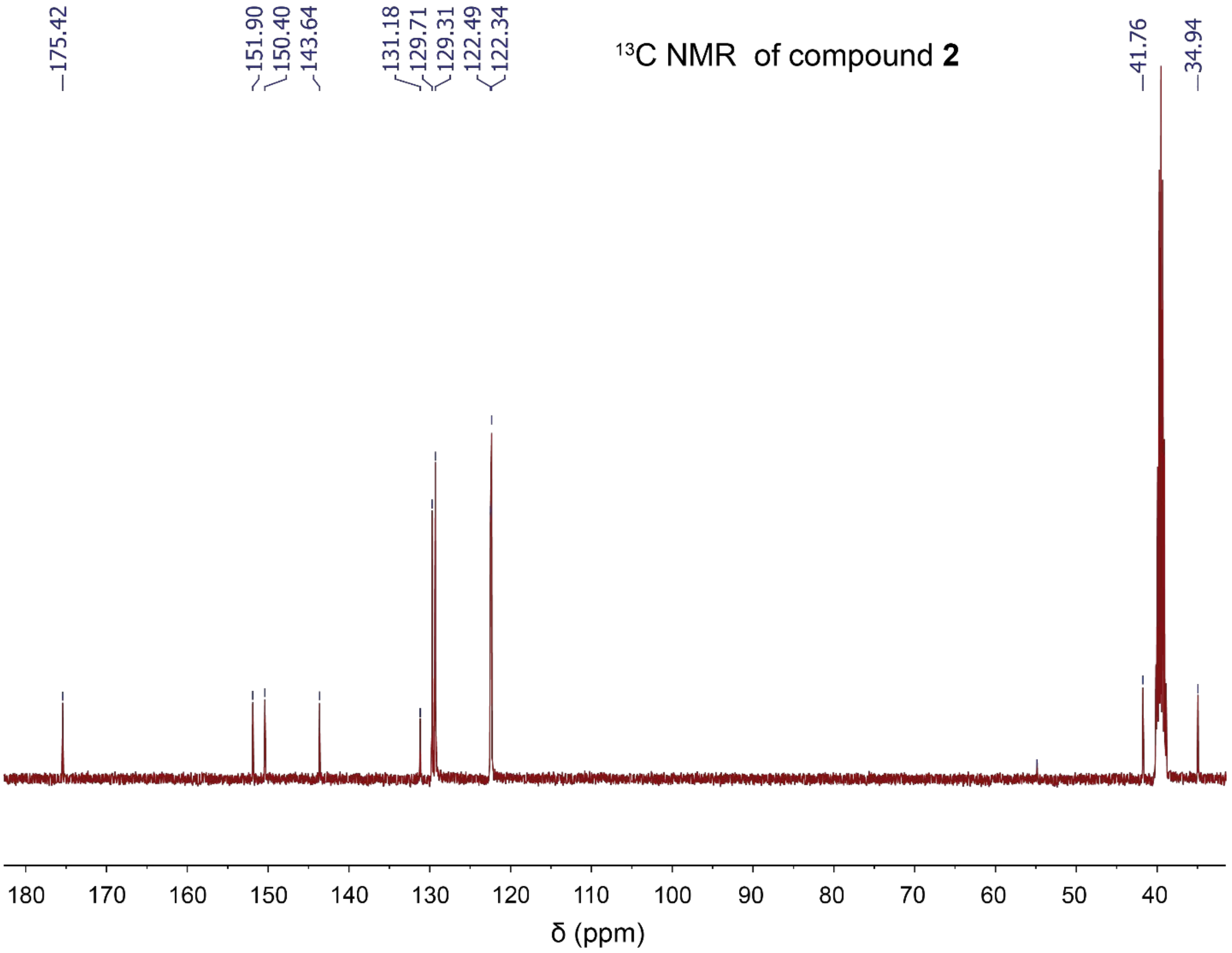

175.42
151.90
150.40
143.64
131.18
129.71
129.31
122.49
122.34
^{13}C NMR of compound **2**
41.76
34.94
180
170
160
150
140
130
120
110
100
90
80
70
60
50
40
δ (ppm)

Scheme 4. Synthesis of compound **3**

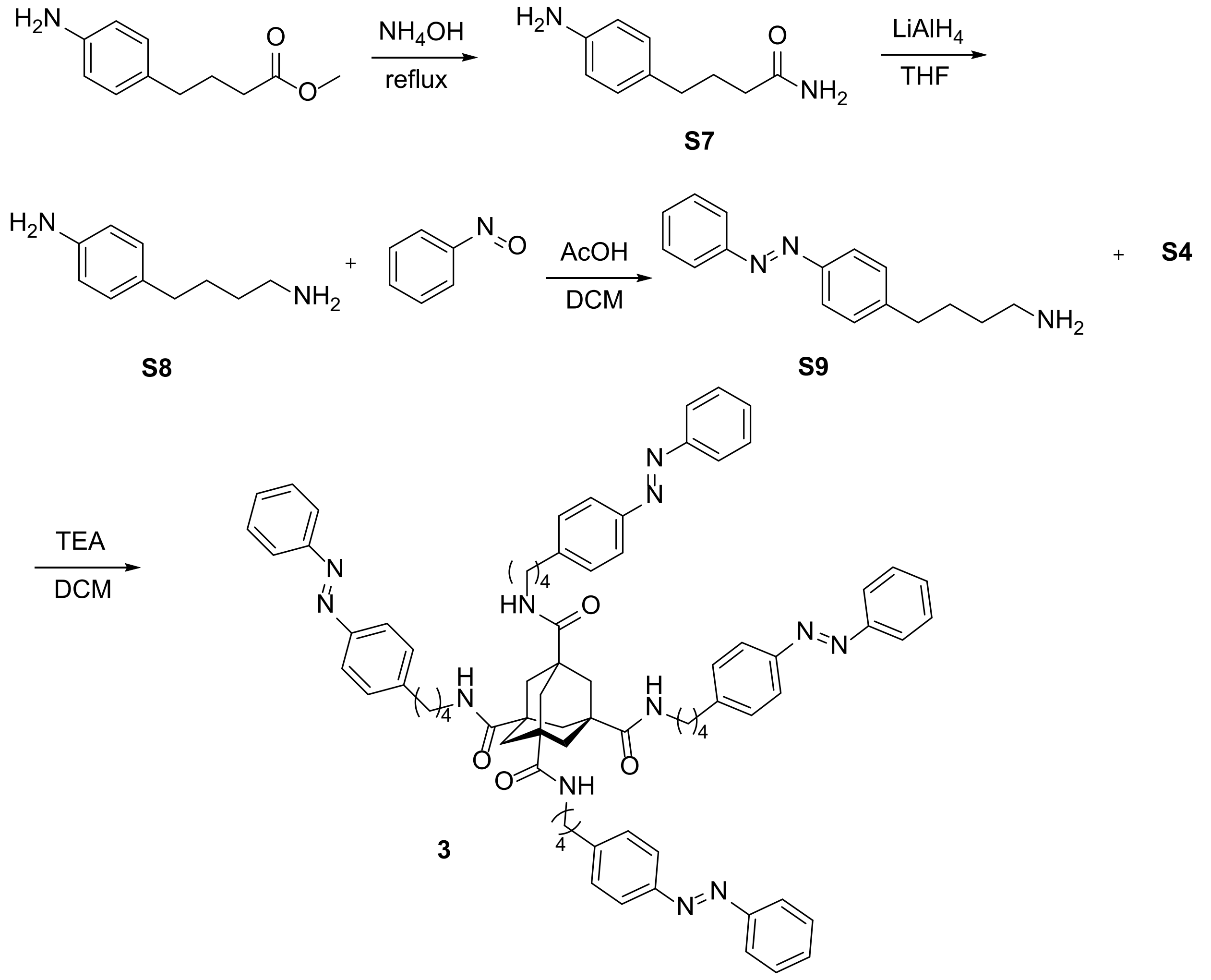

S7: Methyl 4-(4-aminophenyl) butanoate (919.6 mg, 4.8 mmol, 1 eq.) was dissolved in 18 mL methanol, and added to the solution was 36 mL ammonia solution. The reaction mixture was refluxed overnight. The mixture was then cooled to room temperature and evaporated under vacuum to remove residual methanol. The solution was extracted with DCM and washed with water and brine. The organic layer was combined and dried with anhydrous $MgSO_4$. The solvent was removed under vacuum and the pure product was obtained as an off-white solid (304.0 mg, 35.5% yield). ^{1}H NMR (400 MHz, $CDCl_3$) δ 6.98 (d, J=8 Hz, 2H), 6.64 (d, J=8 Hz, 2H), 3.59 (s, 2H), 2.56 (t, J=7.6 Hz, 2H), 2.20 (t, J=7.6, 2H), 1.92 (qui, J=7.2 Hz, 2H) ppm.

S8: **S7** was dissolved in 10 mL dry THF in a 50 mL flame-dried Ar-filled RBF, and lithium aluminium hydride (142.2 mg, 3.7 mmol, 2.0 eq.) in 10 mL dry THF was added dropwise to the

above solution. The reaction mixture was stirred at room temperature for 15 min and then refluxed for 1 h. The reaction was quenched by adding 0.15 mL DI water, 0.15 mL 4M NaOH solution, and an additional 0.45 mL DI water and stirred for another 15 min. The solution was dried by adding sufficient anhydrous $MgSO_4$. After filtration, the solvent was removed by evaporation under vacuum and a yellow oil was obtained as the product (213 mg, 70.1% yield). ^{1}H NMR (400 MHz, $CDCl_3$) δ 6.96 (d, J=8.4 Hz, 2H), 6.63 (d, J=8 Hz, 2H), 3.54 (s, 2H), 2.69 (t, J=7.2 Hz, 2H), 2.51 (t, J=7.6 Hz, 2H), 1.59 (qui, J=6.8 Hz, 2H), 1.46 (qui, J=8 Hz, 2H) ppm.

S9: The synthesis procedure of **S9** was the same as compound **S5**. The product was purified by column chromatography (SiO_2, DCM:MeOH, 95:5) to yield a red solid (226.7 mg, 69.0% yield). ^{1}H NMR (400 MHz, $CDCl_3$) δ 7.91 (d, J=7.2 Hz, 2H), 7.86 (d, J=8.4 Hz, 2H), 7.51 (t, J=6.8 Hz, 2H), 7.46 (t, J=7.2 Hz, 1H), 7.33 (d, J=8.4 Hz, 2H), 3.17 (s, 2H), 2.79 (t, J=7.2 Hz, 2H), 2.71 (t, J=8, 2H), 1.72 (qui, J=7.6 Hz, 2H), 1.58 (qui, J=6.8 Hz, 2H) ppm.

3: The synthesis procedure of compound **3** was the same as compound **1**. And the resulting crude product was purified by column chromatography (SiO_2, pure DCM) to yield a yellow solid (80.5 mg, 32.1% yield). ^{1}H NMR (400 MHz, $CDCl_3$) δ 7.90 (d, J=7.2 Hz, 8H), 7.85 (d, J=8.4 Hz, 8H), 7.50 (t, J=7.6 Hz, 8H), 7.46 (d, J=6.8 Hz, 4H), 7.29 (d, J=8.4 Hz, 8H + $CDCl_3$), 5.81 (t, J=5.6 Hz, 4H), 3.24 (q, J=6Hz, 8H), 2.68 (t, J=7.6 Hz, 8H), 1.90 (s, 12H), 1.65 (q, J=7.2 Hz, 8H), 1.52 (q, J=7.2 Hz, 8H) ppm. ^{13}C NMR (100 MHz, $CDCl_3$) δ175.22, 152.86, 151.24, 145.55, 130.87, 129.22, 129.17, 123.10, 122.86, 42.50, 39.61, 39.56, 35.41, 29.24, 28.46 ppm. HRMS: ESI-MS: *m/z* found [M-H+] for $C_{78}H_{84}N_{12}O_4^+$ 1253.6791 (calcd. 1253.67).

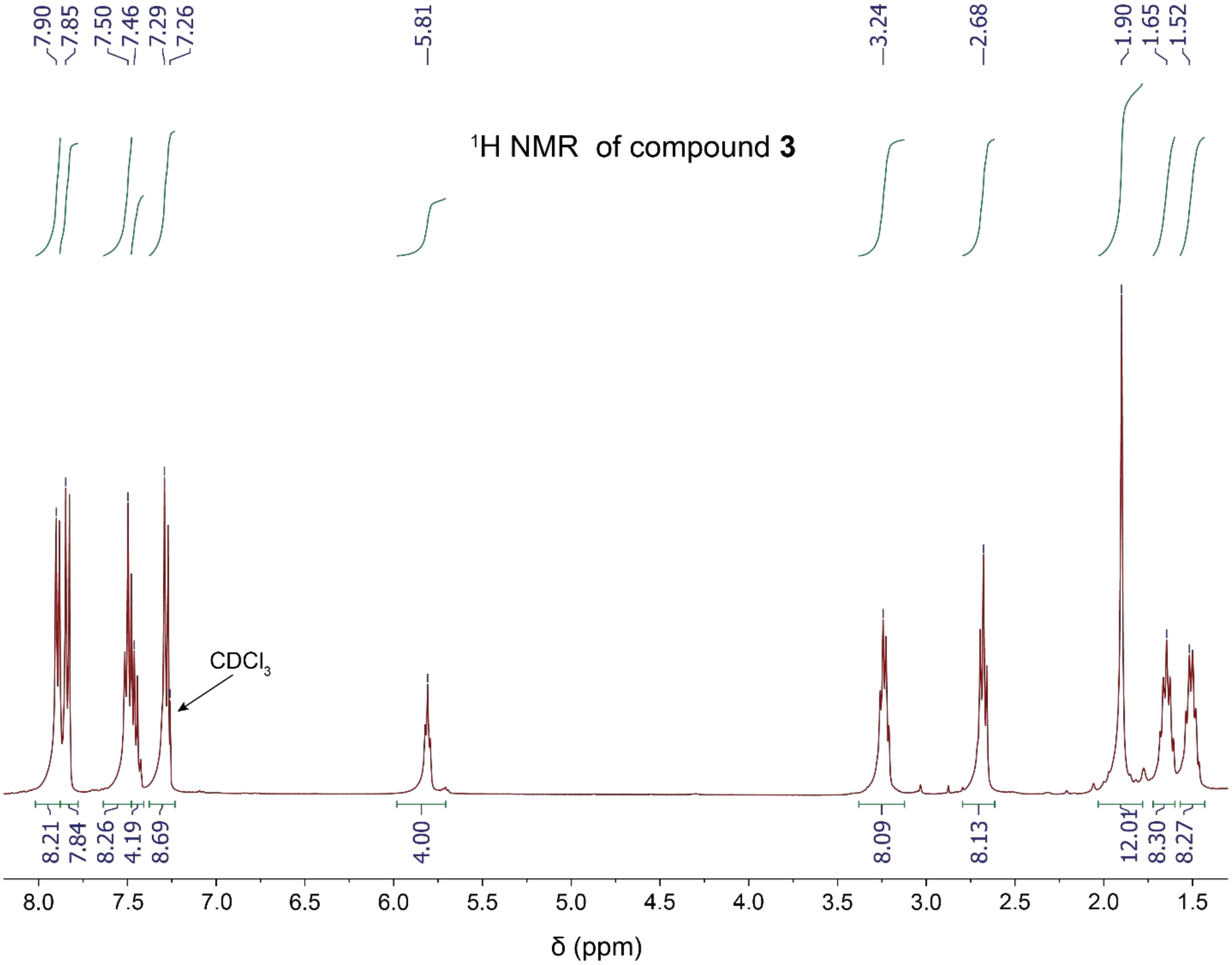

7.90
7.85
7.50
7.46
7.29
7.26
5.81
3.24
2.68
1.90
1.65
1.52
¹H NMR of compound 3
CDCl3
8.21
7.84
8.26
4.19
8.69
4.00
8.09
8.13
12.01
8.30
8.27
8.0 7.5 7.0 6.5 6.0 5.5 5.0 4.5 4.0 3.5 3.0 2.5 2.0 1.5
δ (ppm)

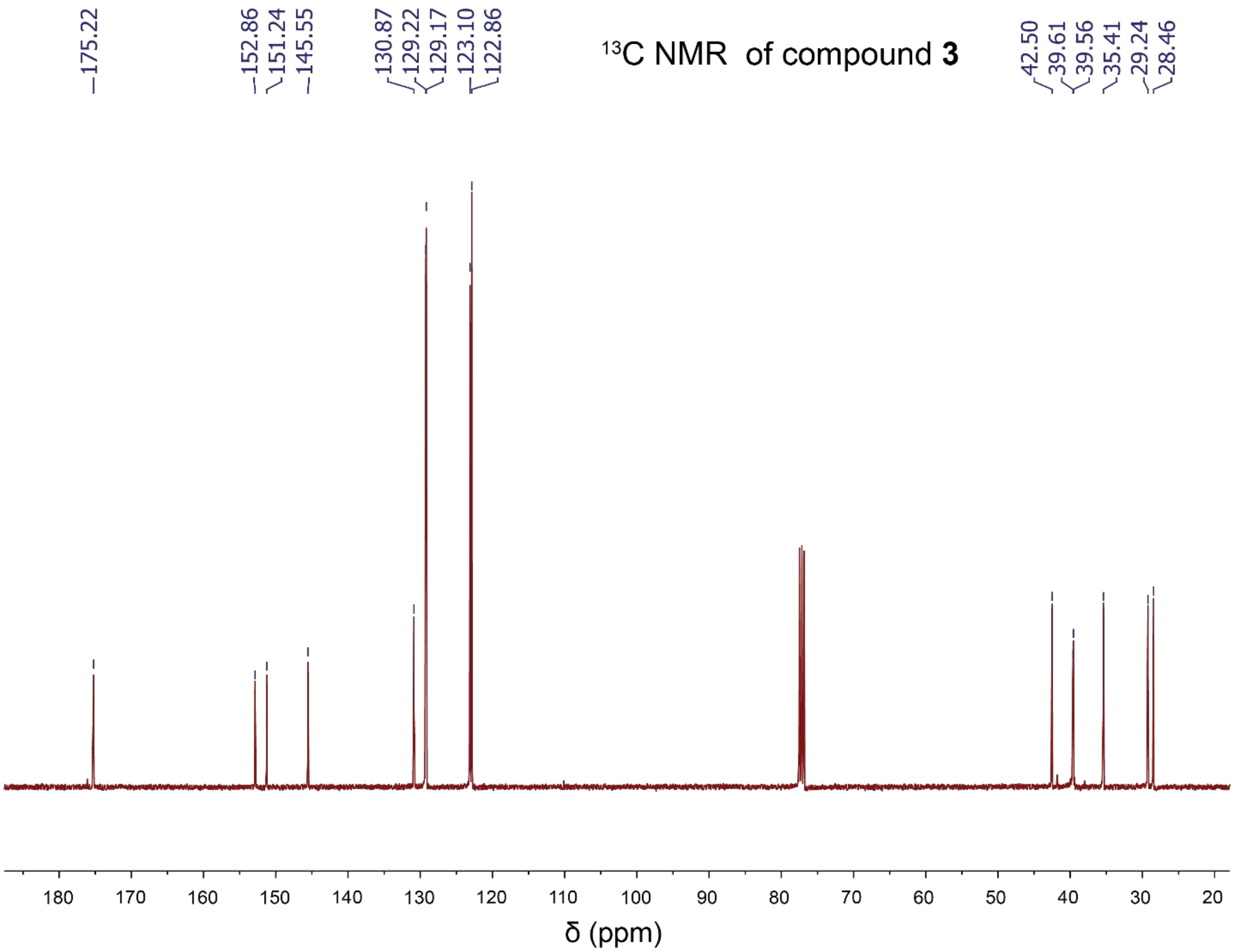

175.22
152.86
151.24
145.55
130.87
129.22
129.17
123.10
122.86
^{13}C NMR of compound **3**
42.50
39.61
39.56
35.41
29.24
28.46
180
170
160
150
140
130
120
110
100
90
80
70
60
50
40
30
20
δ (ppm)

3. Photoswitching Properties in Solution and Solid State

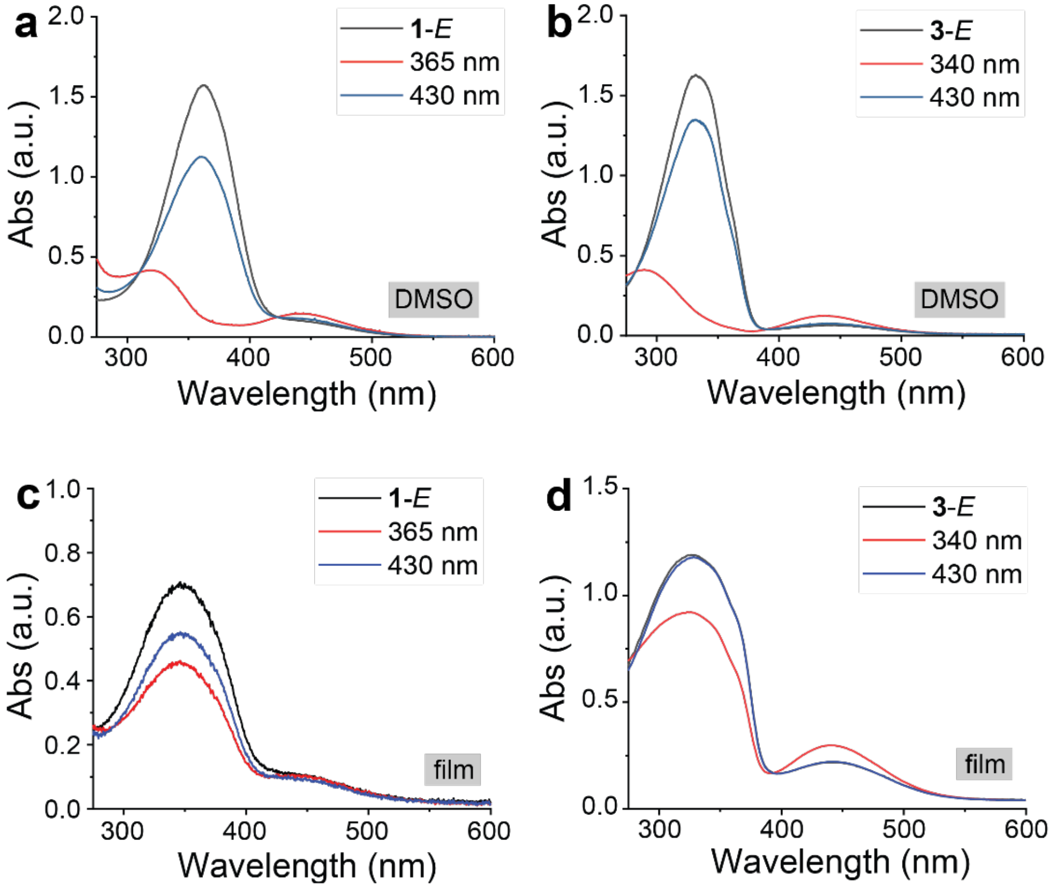

Figure S1. UV-vis absorption spectra of (a) compound **1** and (b) compound **3** as prepared (black), after irradiation at 340/365 nm (red), and 430 nm (blue) measured in DMSO solutions (0.02 mM). UV-vis absorption spectra of (c) compound **1** and (d) compound **3** measured in thin films.

4. Thermal Half-life Measurements in DMSO Solution

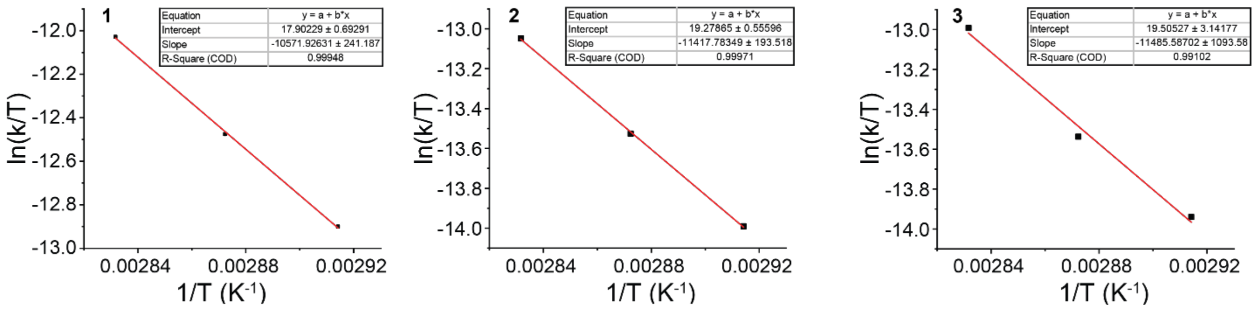

Figure S2. Eyring-Polanyi plots of thermal *Z*-to-*E* isomerization of compounds **1-3** measured in DMSO.

Table S1. Summary of thermal reversion activation energy ($\Delta H^{\ddagger}$, $\Delta S^{\ddagger}$, $\Delta G^{\ddagger}$) and $t_{1/2}$ of *Z* isomers of compounds **1-3** at 298 K.

**Compound**	**1**	**2**	**3**
**$\Delta H^{\ddagger}$ (kJ/mol)**	87.9	96.6	95.9
**$\Delta S^{\ddagger}$ (J/mol*K^{-1})**	-48.7	-32.4	-34.3
**$\Delta G^{\ddagger}$ (kJ/mol)**	102.4	106.3	106.1
**$t_{1/2}$ (days)**	1.1	5.4	5.0

5. Percentage of Isomers at Photostationary State

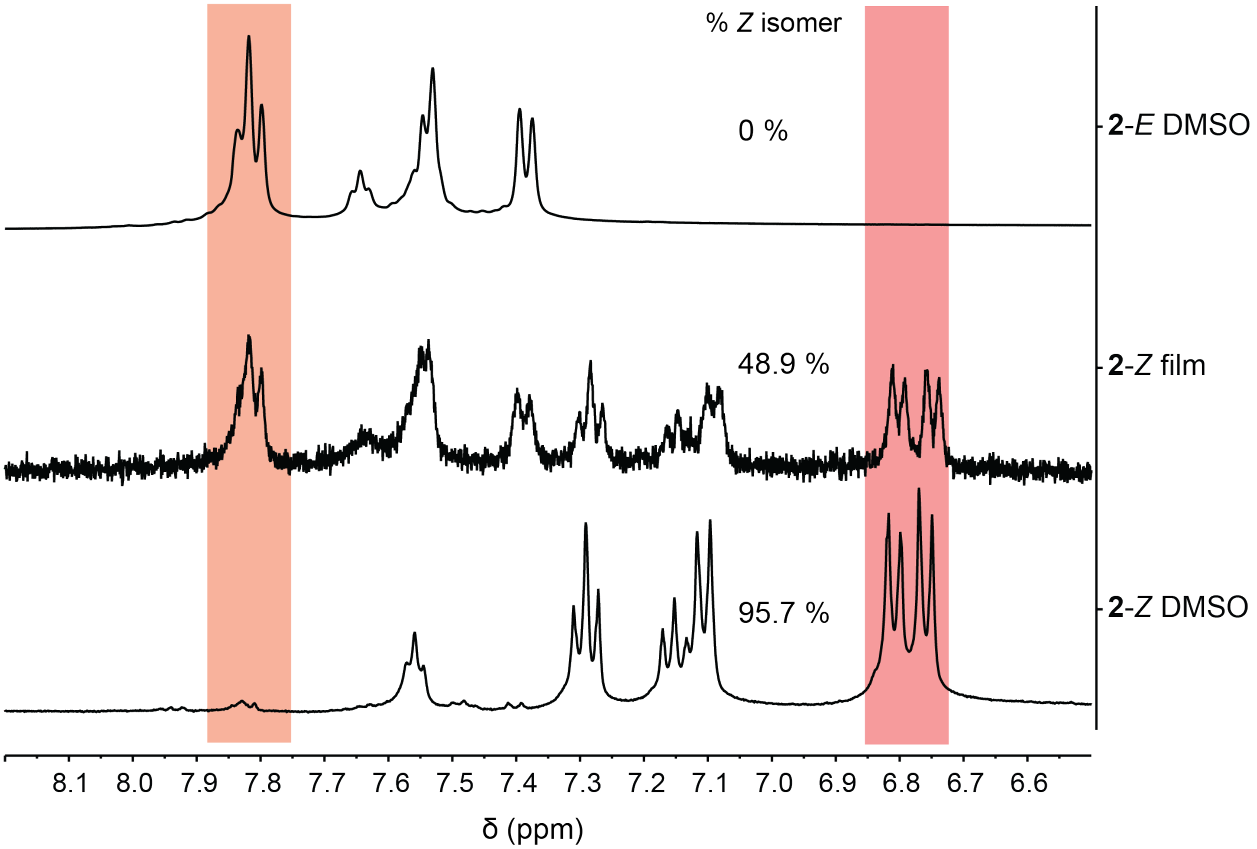

Figure S3. Percentage of **2**-*Z* isomer measured upon 340 nm irradiation in thin film and DMSO, respectively. Peaks used to calculate the percentage of *Z* isomer are highlighted in orange (*E*) and red (*Z*).

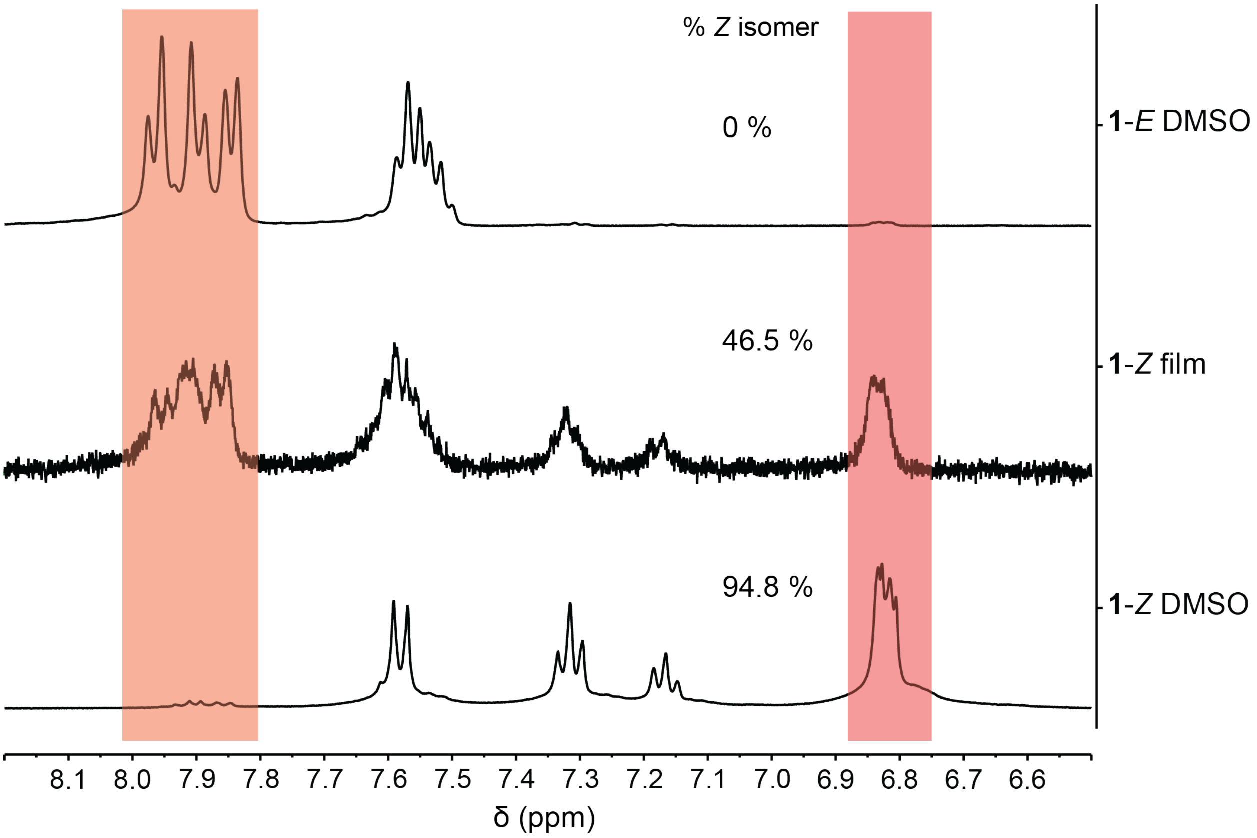

Figure S4. Percentage of **1**-*Z* isomer measured upon 365 nm irradiation in thin film and DMSO, respectively. Peaks used to calculate the percentage of *Z* isomer are highlighted in orange (*E*) and red (*Z*).

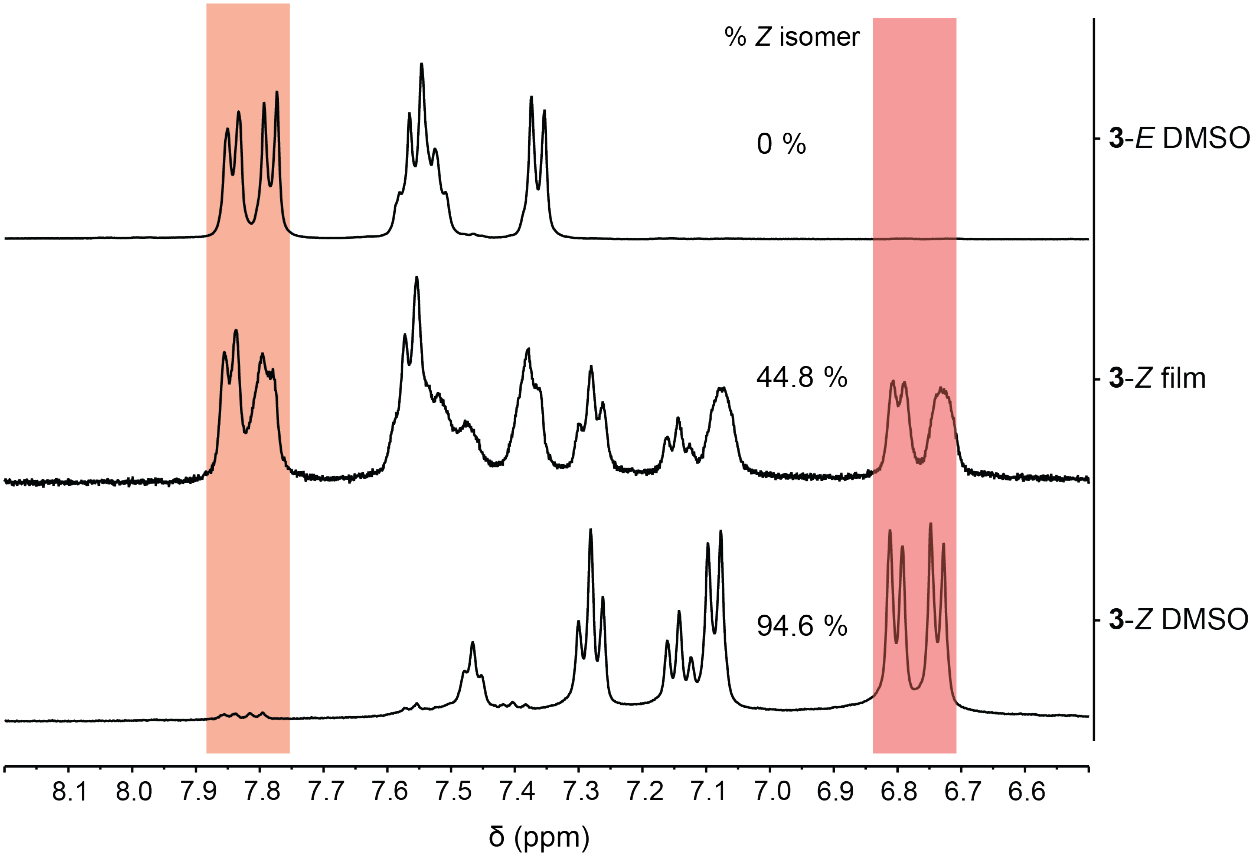

Figure S5. Percentage of **3**-*Z* isomer measured upon 340 nm irradiation in thin film and DMSO, respectively. Peaks used to calculate the percentage of *Z* isomer are highlighted in orange (*E*) and red (*Z*).

6. Thickness Measurements of Thin Films

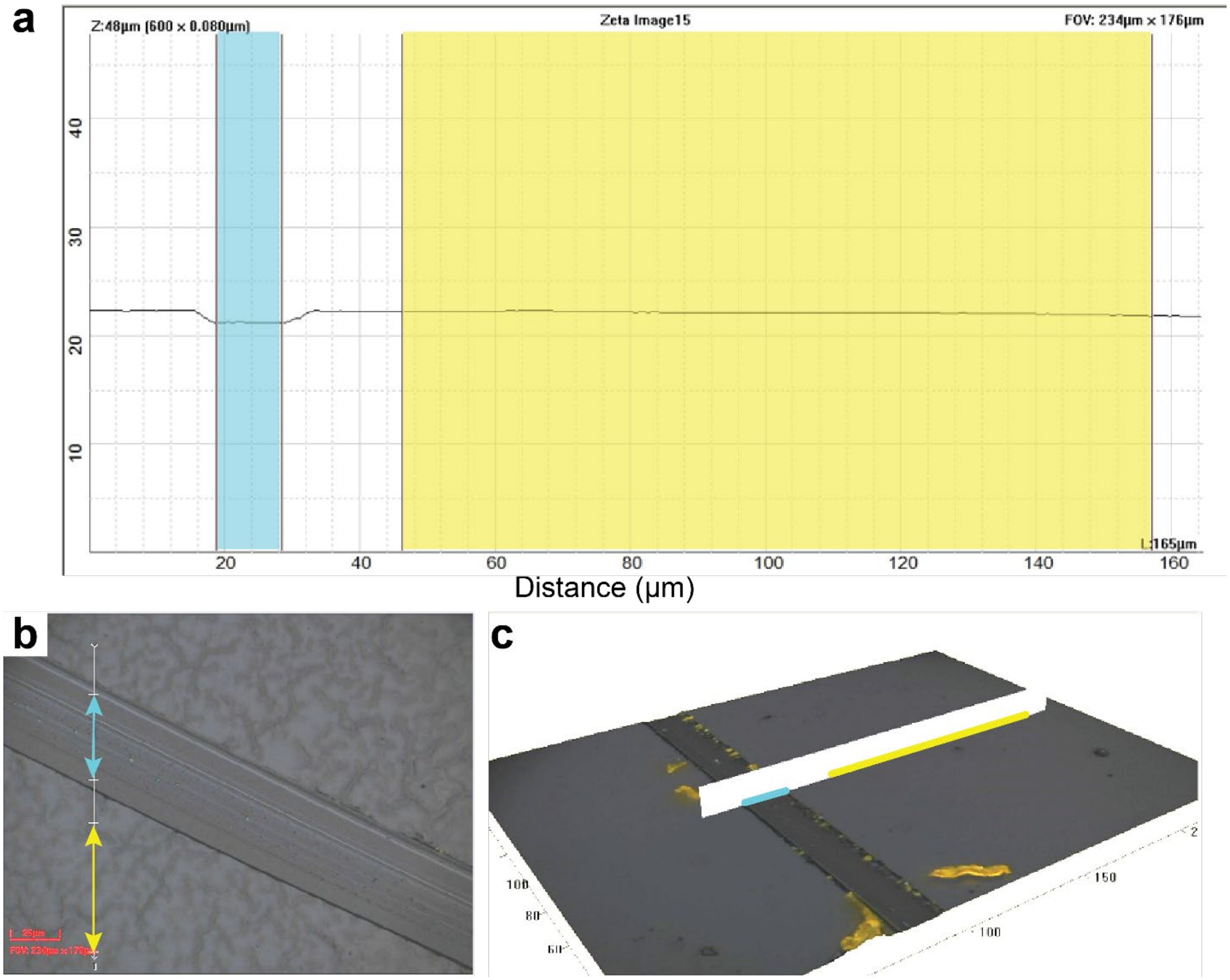

Figure S6. (a) Profile of **1**-*E* film with an average thickness of 0.9 µm. b) Optical microscope image of the measured area. c) 3D topography of the measured area. Glass slide surface area is highlighted in blue while measured sample area is in yellow, and the thickness of the film is the height difference between the two areas.

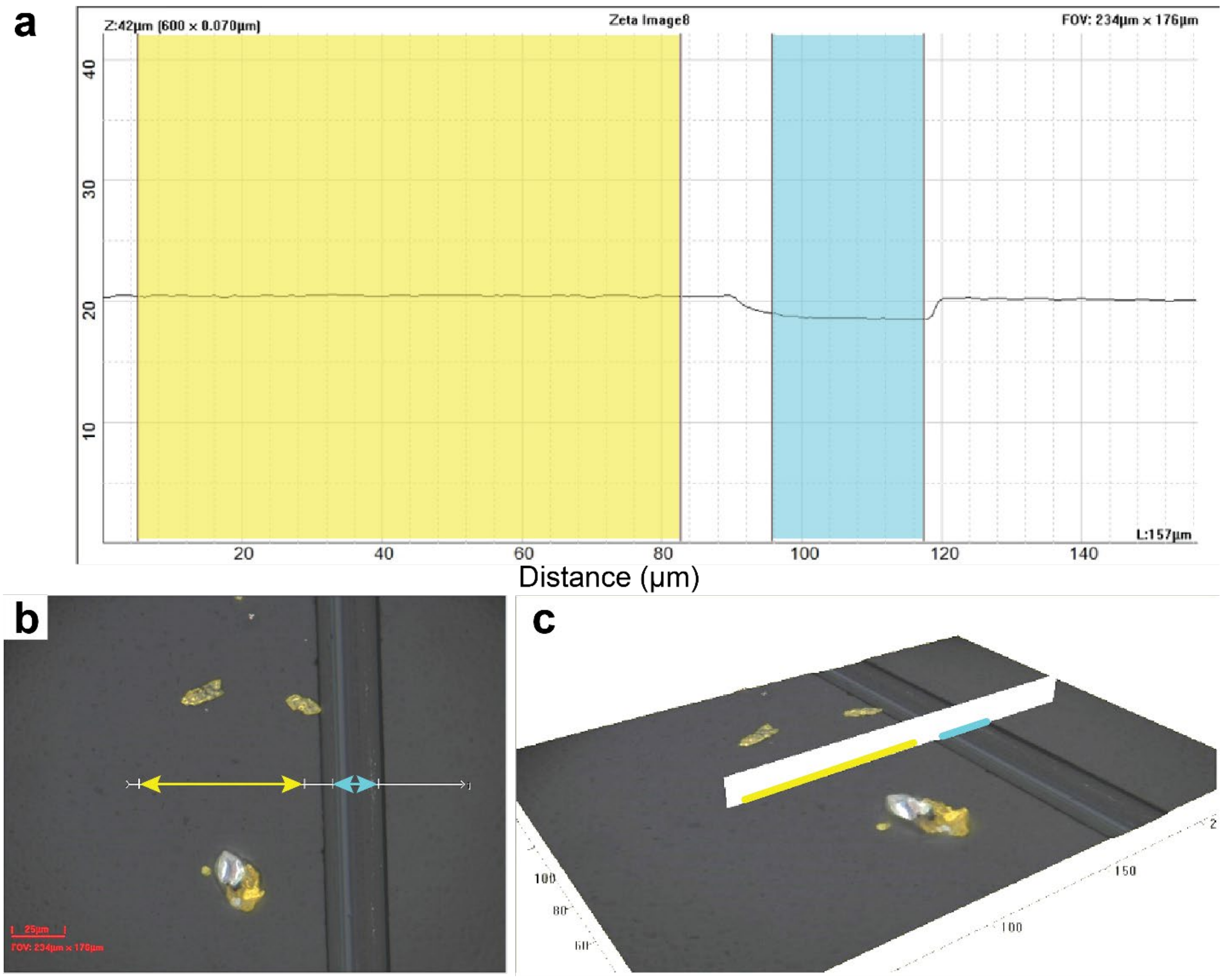

Figure S7. (a) Profile of **2**-*E* film with an average thickness of 1.3 µm. b) Optical microscope image of the measured area. c) 3D topography of the measured area. Glass slide surface area is highlighted in blue while measured sample area is in yellow, and the thickness of the film is the height difference between the two areas.

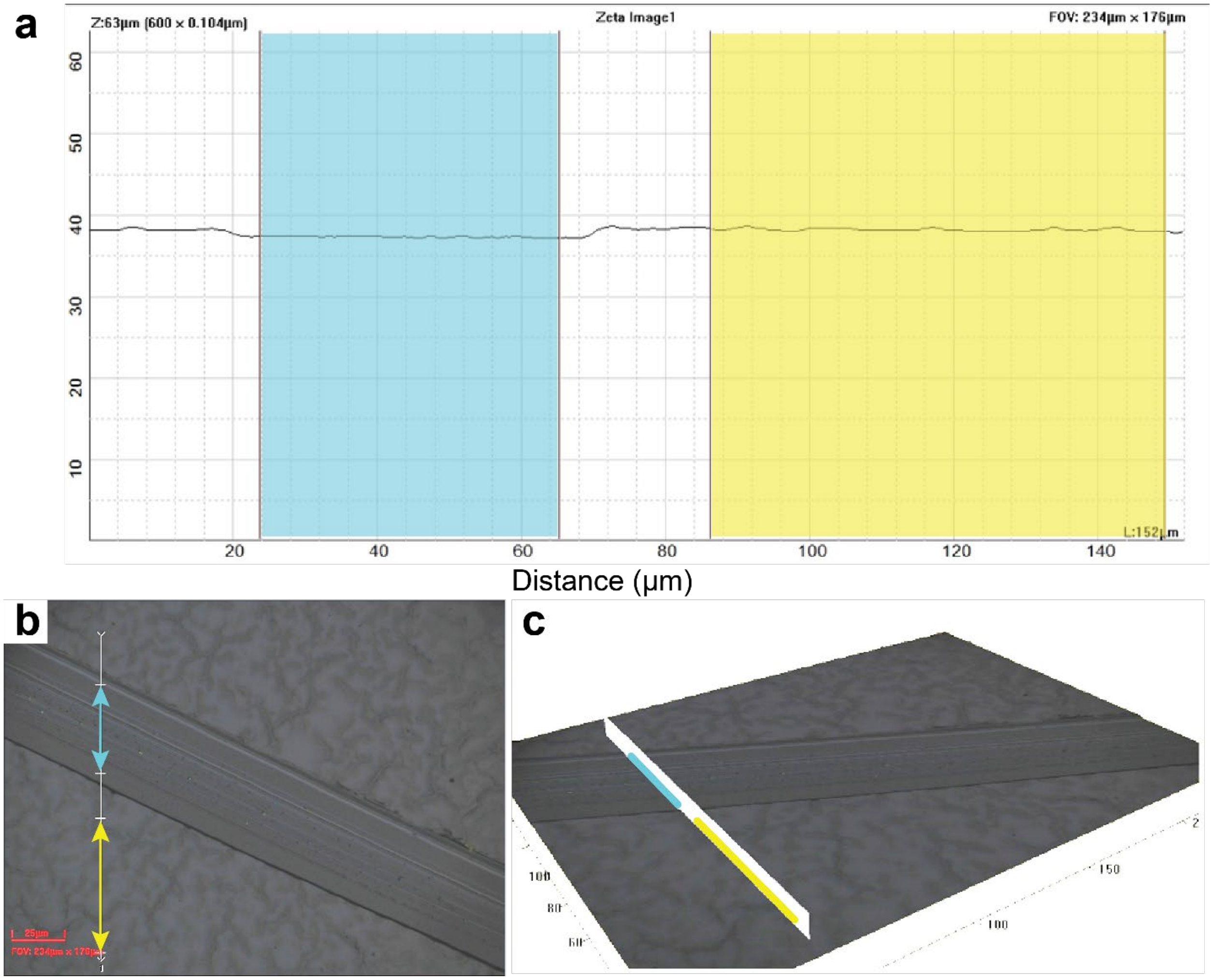

Figure S8. (a) Profile of **3**-*E* film with an average thickness of 1.1 µm. b) Optical microscope image of the measured area. c) 3D topography of the measured area. Glass slide surface area is highlighted in blue while measured sample area is in yellow, and the thickness of the film is the height difference between the two areas.

7. Photoswitching in Solid State (Continued)

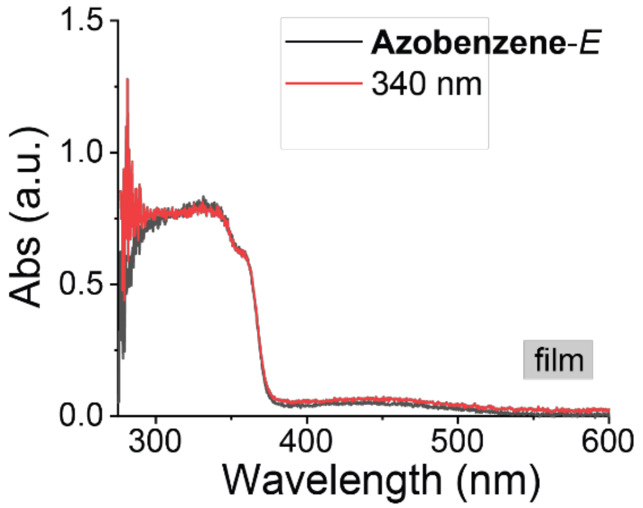

Figure S9. UV-vis absorption spectra of unsubstituted azobenzene measured in thin films.

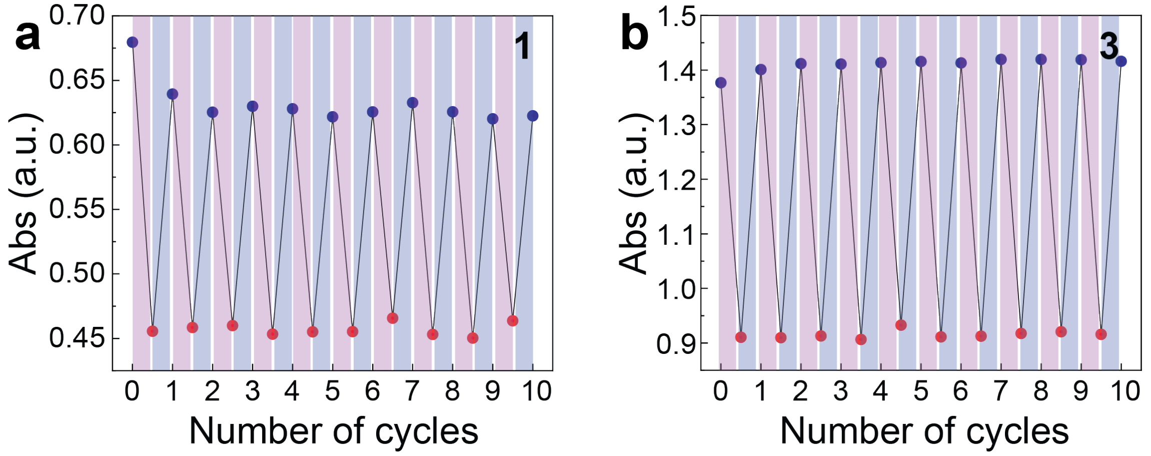

Figure S10. Absorption changes of (a) compound **1** and (b) compound **3** at 362/330 nm upon the repeated irradiation at 365/340 nm (purple filled area) and 430 nm (blue filled area) in thin films. Minor fluctuation of absorbance is attributed to the change in the local concentration of photochromes within the film that is induced during the repeated phase transitions.

8. ^{1}H NMR of 1-*Z* After DSC Measurements.

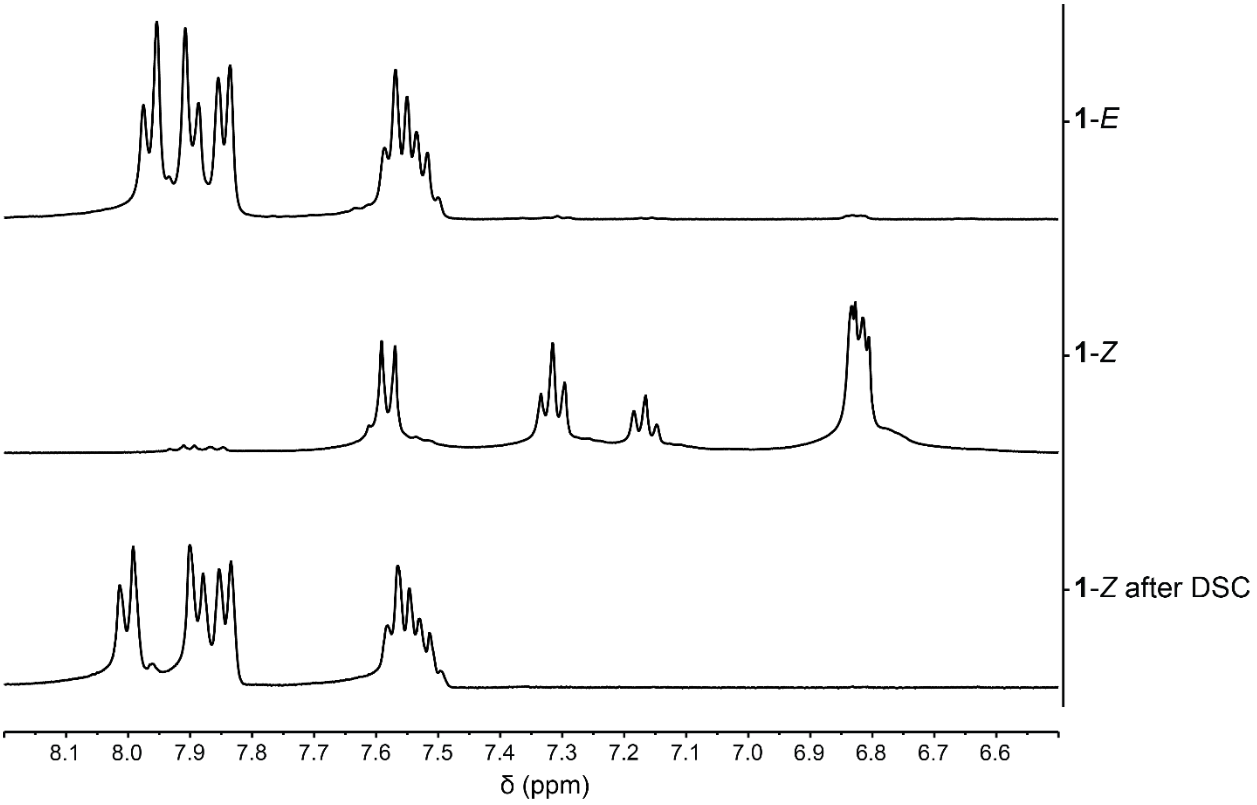

Figure S11. ^{1}H NMR of **1**-*Z* after DSC measurements compared with ^{1}H NMR spectra of **1**-*E* and **1**-*Z*, which confirmed the *Z*-to-*E* thermal reversion after the DSC.

9. DSC Plots

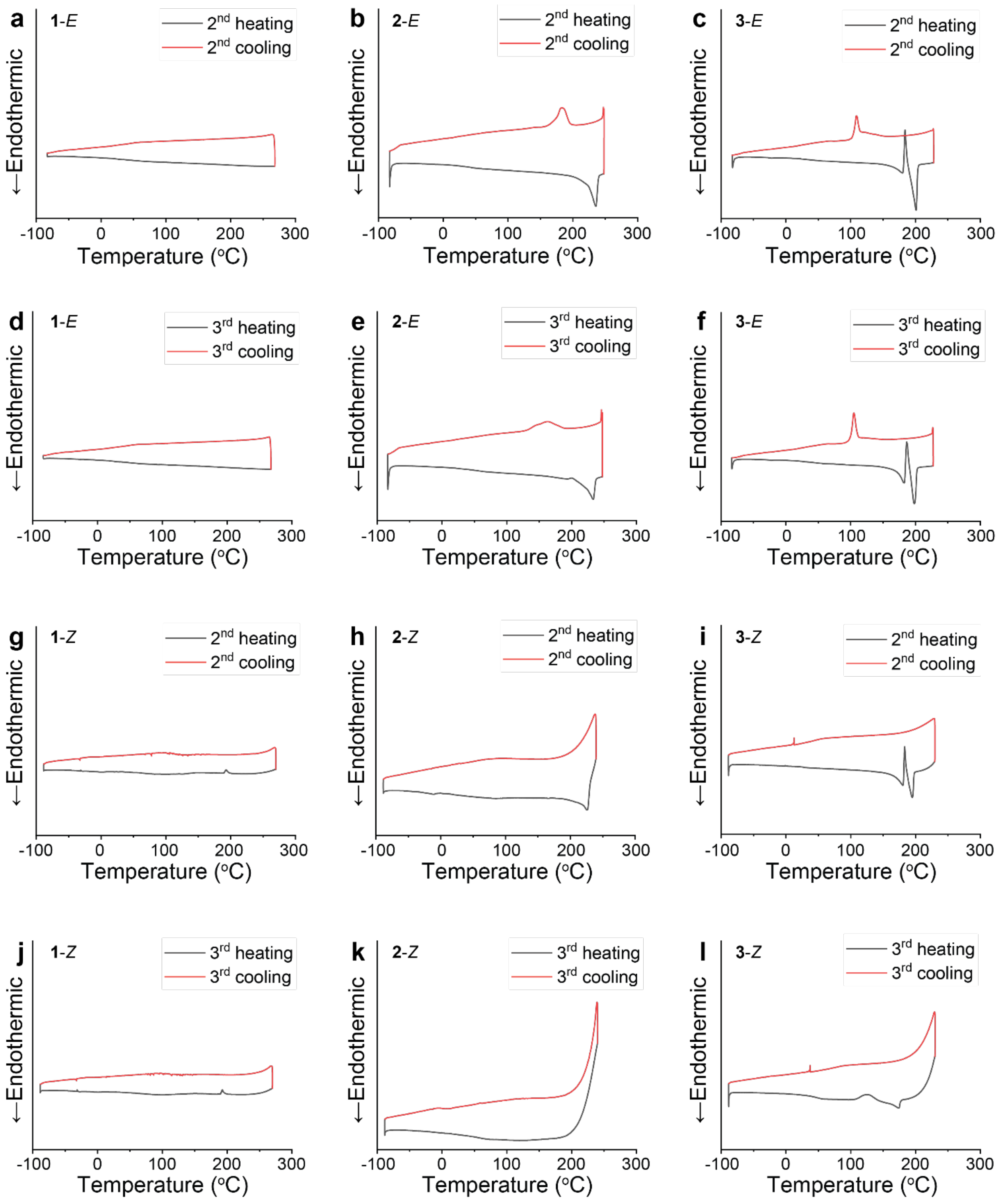

Figure S12. DSC plots of compounds (a, d, g, j) **1**, (b, e, h, k) **2**, and (c, f, i, l) **3** in the *E* and *Z* isomeric forms in their 2nd and 3rd cycles. Thermal decomposition of (h, k) **2**-*Z* and (l) **3**-*Z* is monitored by the exothermic change of curves above 200 °C.

10. PXRD Data

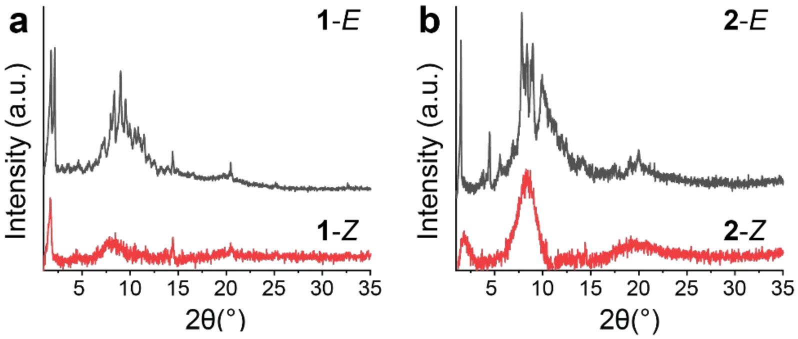

Figure S13. XRD patterns of (a) compound **1** and (b) compound **2** as *E* and *Z* isomers.

11. Gas Adsorption Properties

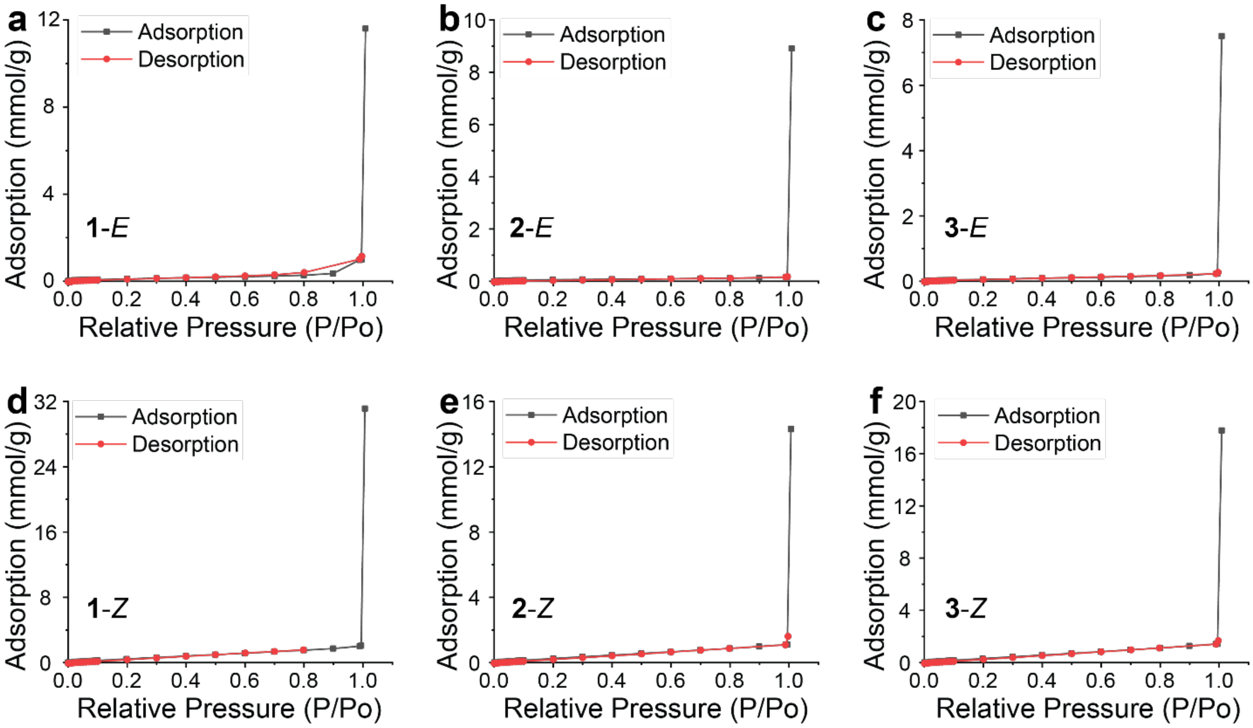

Figure S14. N_2 gas adsorption isotherms of *E* and *Z* isomers of compound **1-3** at 77 K.

Table S2. BET surface area of *E* and *Z* isomers of compound **1-3**.

BET surface area (m^2/g)	**1**	**2**	**3**
E	9.2	5.2	5.9
Z	56.9	33.5	41.5

12. References

(1) Boldog, I.; Reiss, G. J.; Domasevitch, K. V.; Baše, T.; Bräse, S. When Does a Supramolecular Synthon Fail? Comparison of Bridgehead-Functionalized Adamantanes: The Tri- and Tetra-Amides and Amine Hydrochlorides. *Cryst. Growth Des.* **2019**, *19* (9), 5218–5227.

(2) Sayed, S. M.; Lin, B.-P.; Yang, H. Generation of Liquid Crystallinity from a T_d-Symmetry Central Unit. *Soft Matter.* **2016**, *12* (28), 6148–6156.

(3) Lee, G. S.; Bashara, J. N.; Sabih, G.; Oganesyan, A.; Godjoian, G.; Duong, H. M.; Marinez, E. R.; Gutiérrez, C. G. Photochemical Preparation of 1,3,5,7-Tetracyanoadamantane and Its Conversion to 1,3,5,7-Tetrakis(Aminomethyl)Adamantane. *Org. Lett.* **2004**, *6* (11), 1705–1707.

(4) Ernst, C.; Sindlinger, J.; Schwarzer, D.; Koch, P.; Boeckler, F. M. The Symmetric Tetravalent Sulfhydryl-Specific Linker NATBA Facilitates a Combinatorial "Tool Kit" Strategy for Phage Display-Based Selection of Functionalized Bicyclic Peptides. *ACS Omega* **2018**, *3* (10), 12361–12368.
